# Differential receptive field organizations give rise to nearly identical neural correlations across three parallel sensory maps in weakly electric fish

**DOI:** 10.1371/journal.pcbi.1005716

**Published:** 2017-09-01

**Authors:** Volker Hofmann, Maurice J. Chacron

**Affiliations:** Department of Physiology, McGill University, McIntyre Medical Building, Montreal, Québec, Canada; University of California at Berkeley, UNITED STATES

## Abstract

Understanding how neural populations encode sensory information thereby leading to perception and behavior (i.e., the neural code) remains an important problem in neuroscience. When investigating the neural code, one must take into account the fact that neural activities are not independent but are actually correlated with one another. Such correlations are seen ubiquitously and have a strong impact on neural coding. Here we investigated how differences in the antagonistic center-surround receptive field (RF) organization across three parallel sensory maps influence correlations between the activities of electrosensory pyramidal neurons. Using a model based on known anatomical differences in receptive field center size and overlap, we initially predicted large differences in correlated activity across the maps. However, *in vivo* electrophysiological recordings showed that, contrary to modeling predictions, electrosensory pyramidal neurons across all three segments displayed nearly identical correlations. To explain this surprising result, we incorporated the effects of RF surround in our model. By systematically varying both the RF surround gain and size relative to that of the RF center, we found that multiple RF structures gave rise to similar levels of correlation. In particular, incorporating known physiological differences in RF structure between the three maps in our model gave rise to similar levels of correlation. Our results show that RF center overlap alone does not determine correlations which has important implications for understanding how RF structure influences correlated neural activity.

## Author summary

Growing evidence across nervous systems and species shows that the activities of neighboring neurons are not independent but are correlated with one another, which has important implications for neural coding. Such correlations are generally thought to be due to shared input. However, how this shared input is integrated by neurons in order to give rise to correlated activity is not well understood in general. Here we investigated how receptive field structure determines correlations between the activities of electrosensory pyramidal neurons in weakly electric fish. To do so, we used a combination of mathematical modeling of the known antagonistic center-surround RF structure as well as *in vivo* electrophysiological recordings. Our results show that the amount of receptive field center overlap alone is not sufficient to explain experimentally observed neural correlations in general. This is because our experimental data shows that pyramidal neurons with very different amounts of receptive field center overlap display almost identical correlations between their activities. Further, our modeling shows that both receptive field center and surround play important roles in determining correlated activity, such that very different combinations of relative RF surround strength and size can generate nearly identical correlations between neural activities. We discuss the implications of our results for sensory processing.

## Introduction

Encoding sensory stimuli and integrating this information into suitable behavior is a highly complex task that is continuously solved by our nervous system. Nonetheless, the mechanisms by which this is achieved (i.e. the neuronal code) remain poorly understood. There is a growing body of evidence that perception and behavior are determined by the integrated activity of large neural populations [1]. However, our understanding of such population codes is complicated by the fact that neural responses are often correlated and not independent of one another. Correlations between the spike counts of neurons have traditionally been separated into two components: signal correlations (i.e., correlations between the average responses of neurons) which are due to similarities in stimulus-response properties, and noise correlations (i.e., correlations between the trial-to-trial variabilities of the neural responses) that are due to shared synaptic input [2]. Noise correlations have been observed ubiquitously across systems and species [1,3] and there is general agreement that they will strongly influence sensory processing [2,3]. However, the fact that these strongly depend on factors such as the organism’s behavioral state [1,4] as well as stimulus statistics [5–8] greatly complicates understanding their impact on coding in general.

Much effort has gone into the investigation of how shared neural input gives rise to noise correlations. Modeling studies have shown that the balance between excitatory and inhibitory inputs is critical towards determining the sign and magnitude of noise correlations [9,10]. Other studies have instead focused on understanding how the spiking nonlinearity influences correlation transfer [11]. In contrast, much less is known about how receptive field (RF) structure influences correlated activity [12,13]. Thus, studies performed in sensory systems with well-characterized RF structure and anatomical correlates can aid our understanding as to how RF structure and organization influences neural correlations. For this we use the weakly electric fish *Apteronotus leptorhynchus*, which benefits from a well-characterized anatomy and physiology [14–17].

**Fig 1:**
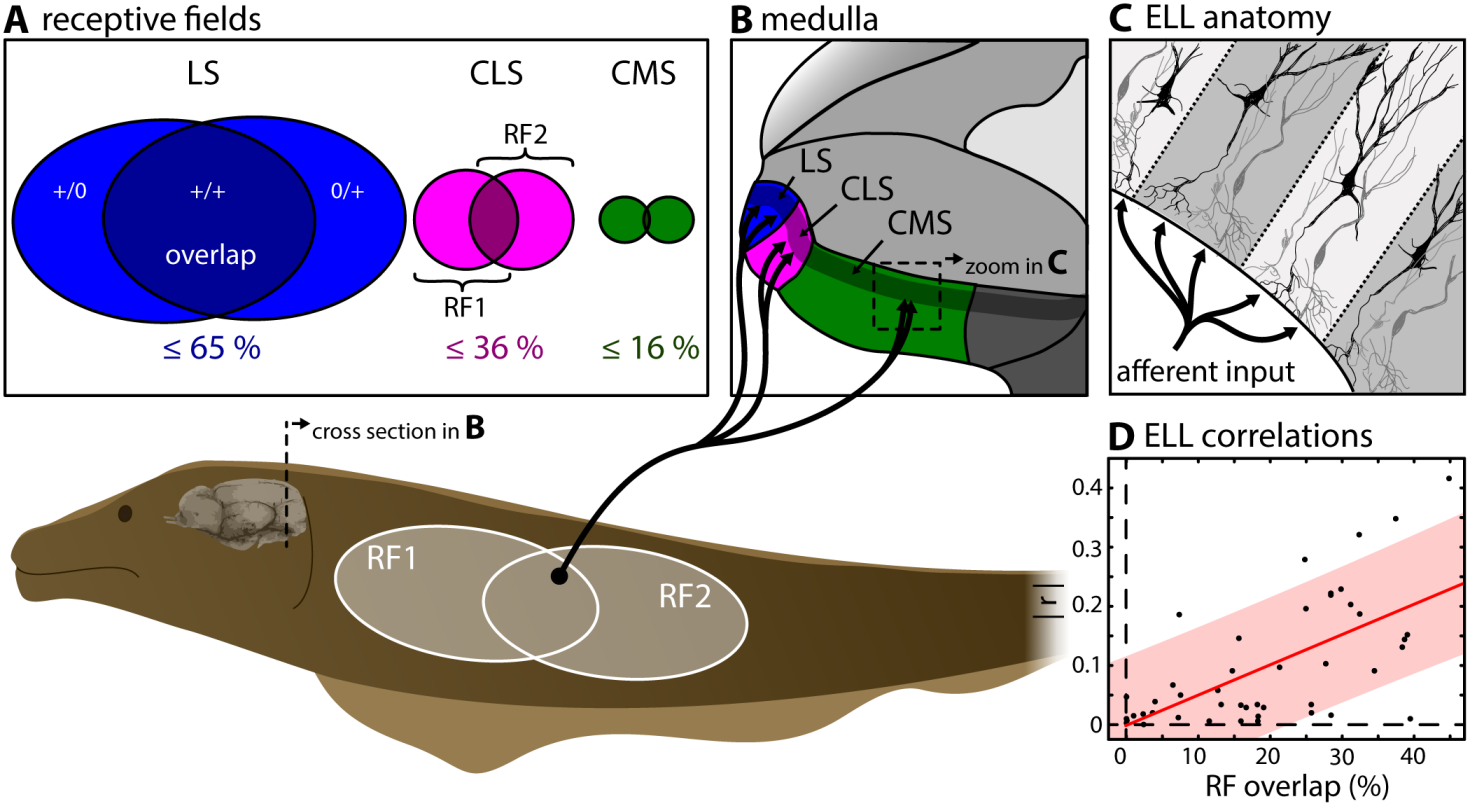
Afferent electrosensory projections trifurcate to three parallel maps in the ELL. **(A)** Peripheral input from cutaneous electroreceptors (p-units) to pyramidal cells in the electrosensory lateral line lobe (ELL, see B & C) is organized in receptive fields (RF) that differ in size and overlap [30] between 3 parallel segments of the ELL. **(B)** Electroreceptors on the skin trifurcate into each of the three parallel ELL segments (LS: lateral segment (blue); CLS: centro-lateral segment (magenta); CMS: centro-medial segment (green)). **(C)** The pyramidal neurons within each segment are the sole output cells and are organized in columns. **(C)** Correlations between the activities of pyramidal cell pairs in CLS were shown previously to increase linearly as a function of RF overlap (data reproduced from [7]).

These fish sense amplitude modulations of a self-generated electric organ discharge (EOD) through an array of electroreceptors distributed over their body [18–21]. While objects with a conductivity different than that of the surrounding water (e.g., plants, rocks, prey) cause spatially localized amplitude modulations, interaction between the EODs of conspecifics will instead give rise to spatially diffuse amplitude modulations [22]. Sensory afferents project to the medullary electrosensory lateral line lobe (ELL) where each axon trifurcates and synapses onto pyramidal neurons within three parallel and somatotopically organized sensory maps within the lateral, centro-lateral and centro-medial segment (LS, CLS & CMS, Fig 1A–1C) [23]. Pyramidal cells within each map are differentially tuned [24,25] and mediate appropriate behavioral responses to stimuli occurring in different contexts [26] (see [16] for review). Pyramidal cells in all maps display an antagonistic center-surround receptive field (RF; the area of sensory space within which impinging stimuli cause a neural response) organization, similar to that of retinal ganglion cells in the visual system [22,27–29]. However, both anatomical and physiological studies have shown substantial differences in the RF structure of pyramidal cells across the maps both in terms of size and % overlap (LS: ≤ 65%; CLS: ≤ 36%; CMS ≤ 16%; Fig 1A) [28,30]. These differences are expected to give rise to increasing levels of correlations between pyramidal cell activities when going from CMS to LS.

To date, most studies have assumed that noise correlations between pyramidal cell activities are negligible (e.g., [31,32]), in part because anatomical results have shown low RF % overlap values in both CMS and CLS [30]. Only a few studies have recorded the simultaneous responses of multiple electrosensory neurons and quantified correlations [7,8,33–35]. These have shown that pyramidal cells can display significant correlation coefficients ranging between 0 and 0.5 that are at least partially due to RF overlap (Fig 1D) [7,8]. However, these studies have only considered recordings from CLS and LS. Thus, no study to date has systematically quantified and compared correlations between the activities of electrosensory pyramidal neurons across all three ELL maps.

Here, using a combination of mathematical modeling and *in vivo* electrophysiology, we investigated how receptive field structure influences correlations between the activities of pyramidal neurons across the three parallel ELL maps. Experimental data reveals that, contrary to predictions that correlation magnitude increases from CMS to LS based on increasing amounts of RF center overlap, correlations between pyramidal cell pairs are actually not significantly different from one another on average across the three ELL maps. To explain this surprising result, we modeled the contributions of both RF center and surround to determine correlated activity. We found that very different combinations of RF center-surround relative gain and size can give rise to similar correlations because of similar relative amounts of positively and negatively correlated inputs. Importantly, including previously published experimental data on differences between the RF center-surround organization across the maps gave rise to similar average levels of correlations. Our model thus predicts that the similar levels of correlations observed experimentally arise because cells receive similar relative levels of positively and negatively correlated inputs, despite very different RF center-surround organizations.

## Results

### Modeling RF center input alone predicts that correlations are proportional to RF center overlap

In this study, we focused on understanding how differences in RF center size and overlap influence correlated ELL pyramidal cell activity. To do so, we focused on correlations between neural activities measured under baseline condition (i.e., in the absence of stimulation). These are key determinants of noise correlations under stimulation because baseline correlations can be thought of as the limit towards which noise correlations tend to as stimulus intensity goes to zero (see Methods).

**Fig 2:**
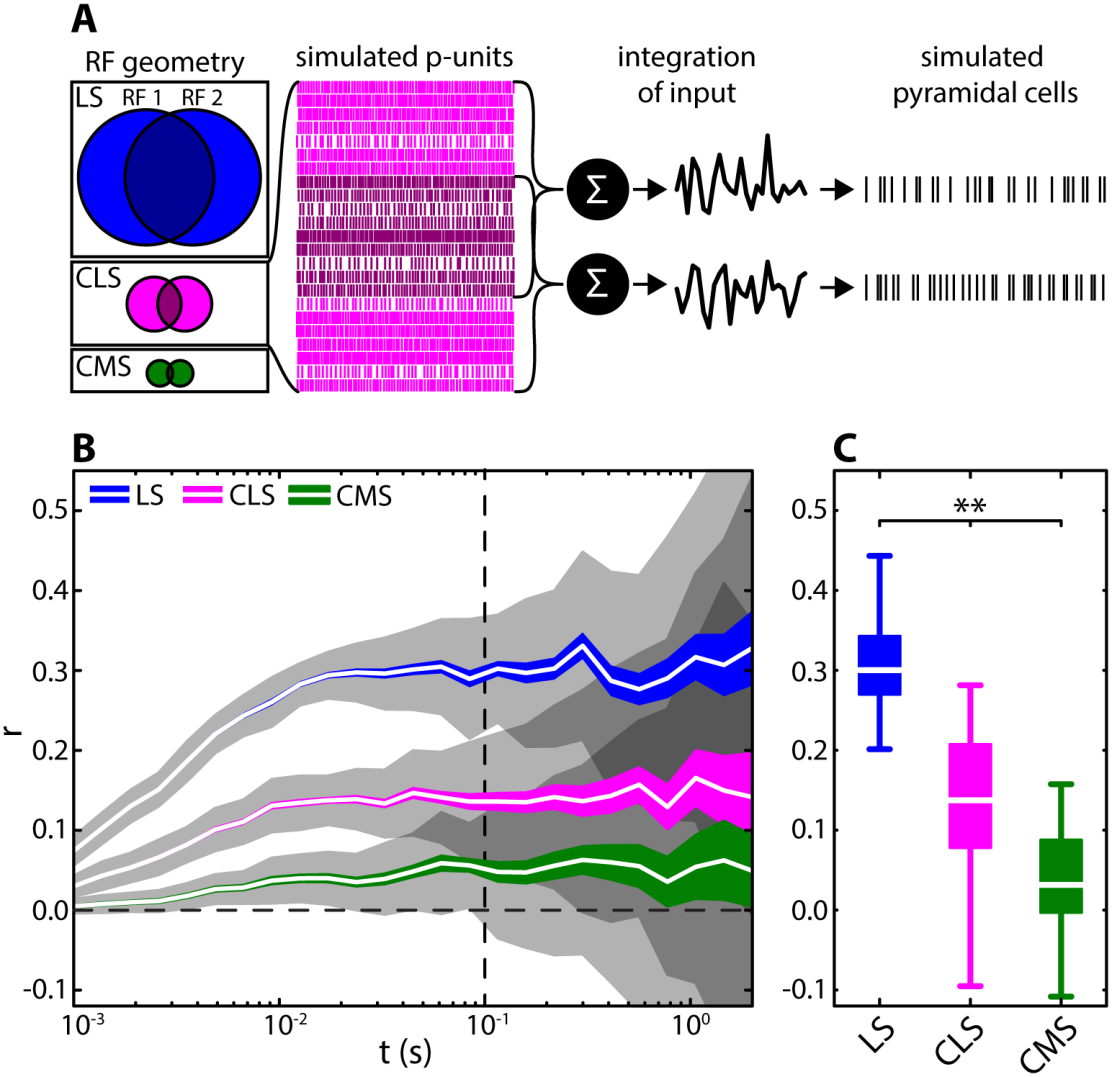
Effect of RF center overlap on correlations in the three parallel ELL segments. **(A)** We modeled differences in RF center size and overlap by varying the number of afferents converging onto pyramidal cells (LS: blue; CLS: magenta; CMS: green). We used phenomenological and accurate models of peripheral afferent activity and summed their spiking activities to obtain two input signals. The input signals to both pyramidal cells consisted of independent (bright magenta) and common (dark magenta) afferent populations. The signals served as inputs into two model ELL pyramidal neurons. We computed correlations between the spiking outputs of these two model ELL pyramidal neurons on different timescales t ranging between 10^−3^ s and 1.5 s. RFs consisted of 640, 105, and 25 afferents for LS, CLS, and CMS, respectively. RF overlap were 56.9, 33.3, and 13.2% for LS, CLS, and CMS as per anatomical data [30] respectively. **(B)** Correlations between the spiking activities of the model ELL pyramidal neurons as a function of time window for the three different segments (see legend). Shown are mean (white lines), SEM (colored areas) and STD (gray shaded areas) across 250 random model realizations for 20 s duration. The correlation coefficients increased with time window size in a non-linear fashion. The overall correlation magnitude strongly decreased from LS to CMS (at t = 100 ms: median LS: 0.32, range: -0.10–0.74; CLS: 0.17, -0.27–0.77; CMS: 0.08, -0.43–0.59). **(C)** Distributions of correlation coefficients for the three ELL segments for t = 100 ms (see vertical dotted line in B). At this time scale the means of the distributions differed significantly (Kruskal-Wallis dF = 2; Chi^2^ = 97.48; p = 6.8 · 10^−22^). Qualitatively similar results were obtained for other time windows (e.g.: t = 10 ms; Chi^2^ = 130.8; p = 3.8 · 10^−29^; or t = 1 s; Chi^2^ = 22.73; p = 1.16 · 10^−5^).

We used a simple yet accurate phenomenological model of receptor afferent activity [36,37] to simulate a population of peripheral afferents synapsing onto pyramidal cells. Model parameters were adjusted to reproduce experimentally observed heterogeneities in the baseline activities of afferents [38] (see Methods). Afferent activities were then used as input to two ELL pyramidal neurons that were modeled using the leaky integrate-and-fire formalism (see Fig 2A). We initially only included the contribution of the RF center in the model and adjusted both RF size and % overlap according to the available anatomical data [30]. Specifically, we used 640 model afferents as input for LS center, 105 model afferents as input for CLS center, and 25 model afferents as input for CMS center. To further mimic the different degrees of RF overlap, we adjusted the number of common afferent activities within two different populations that serve as input to two pyramidal cells. The % of shared input was high in LS (57%), intermediate in CLS (33.4%), and low in CMS (13%). Correlations were calculated as spike-count correlations at different timescales (see Methods) from the simulated paired spiking output of the ELL LIF model.

Our modeling results predict that the different amounts of RF center overlap for pyramidal neurons in the three different ELL maps will give rise to very different levels of correlated activity (Fig 2B). Indeed, correlation coefficients obtained from our model were always highest for LS, intermediate for CLS and lowest for CMS (Fig 2B & 2C).

### Experimental data reveals that correlations between ELL pyramidal cell activities are on average nearly identical in all ELL segments

**Fig 3:**
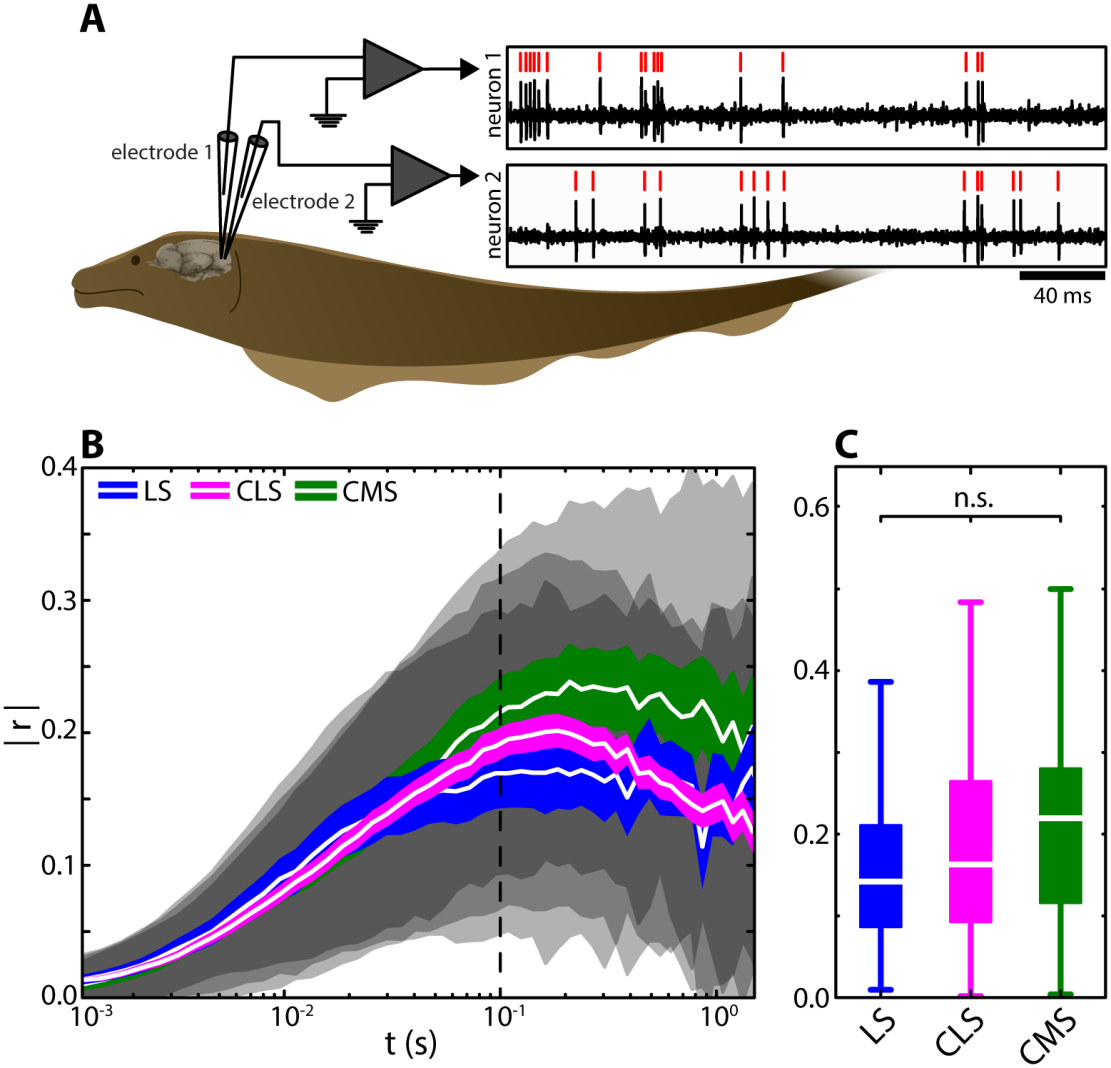
Correlations between ELL pyramidal cell activities are nearly identical across the ELL segments. **(A)** Simultaneous extracellular recordings of baseline activity (i.e. in the absence of stimulation) from pairs of pyramidal cells were obtained in all three ELL segments. Recordings were performed using two metal-filled micropipettes inserted into the pyramidal-cell layer of the respective ELL map (mean recording duration: 105 s; range: 39–318 s). **(B)** Absolute spike-count correlation coefficient for LS (blue), CLS (magenta), and CMS (green) as a function of the time window t. Shown are mean (white lines), SEM (colored areas) and STD (gray shaded areas) correlation coefficients across the populations of pairs recorded in each segment (LS: N = 23; CLS: N = 108; CMS: N = 24). The correlation coefficients obtained for the three segments were similar and thus largely overlapped with one another. (at t = 100 ms: median LS: 0.14, range: 0.009–0.44; CLS: 0.16, 0.001–0.48; CMS: 0.22, 0.003–0.5). **(C)** Population-averaged absolute correlation coefficient for t = 100 ms (see vertical dotted line in B). At this time scale, the means of the distributions were not significantly different from one another (Kruskal-Wallis; df = 2; Chi^2^ = 1.66; p = 0.44). Qualitatively similar results were obtained for other time windows (e.g.: t = 10 ms; Chi^2^ = 1.64; p = 0.44; or t = 1 s; Chi^2^ = 2.78; p = 0.25).

To test our modeling predictions, we simultaneously recorded spontaneous activity from pyramidal cell pairs within each of the three ELL maps (Fig 3A). For each pair, correlations between spike trains were computed in the same way as for the model. Surprisingly, and in contrast to our model predictions, we found that correlations were identical across the ELL segments (Fig 3B). Indeed, pyramidal cell pairs across the three ELL segments displayed on average similar levels of correlated activity and the distributions of correlation coefficients obtained from each map were not significantly different from one another across multiple timescales (Fig 3B & 3C).

We found correlation estimates computed from independent pyramidal cell pairs recorded in different individual animals were not significantly different than those computed using our entire dataset (see Methods and S1 Fig). Further, similar levels of correlated activity were obtained for pairs that were recorded on different electrodes (different-electrode pairs) as compared to pairs that were recorded on the same electrode (same-electrode pairs, S2 Fig), indicating that our measurements of correlated activity do not depend on the recording technique used. Our results show further that the large variability of correlation coefficients within each map originates from heterogeneities between pairs. This is because the time varying correlation coefficients computed for a given pair displayed much lower variance than that seen across pairs (S3 Fig). We also note that differences between the baseline firing rates of neurons within a given pair did not influence magnitude of variability of correlation estimates (S4 Fig). Previous studies have shown that there are two types of pyramidal cells: ON-type cells that are excited and OFF-type cells that are inhibited by increases in EOD amplitude, respectively [39]. To investigate how pyramidal cell type influences correlated activity, we segregated pyramidal cell pairs into three categories: ON-OFF, ON-ON, and OFF-OFF. Overall, we found that opposite type pairs (i.e., ON-OFF) on average displayed negative correlations, while same type pairs (i.e., ON-ON and OFF-OFF) displayed positive correlations on average (S5 Fig). All pair types displayed similar levels of correlation magnitude on average (S5 Fig, see black lines). We found that, at small timescales, correlations were weakly related to firing rate (time windows < 75 ms; S6A, S6B, S6D & S6E Fig), which is consistent with results obtained in other systems [11,40]. However, we found that there was no such relationship for larger time windows (S6C, S6D & S6E Fig).

### Effects of receptive field center-surround organization on correlations

**Fig 4:**
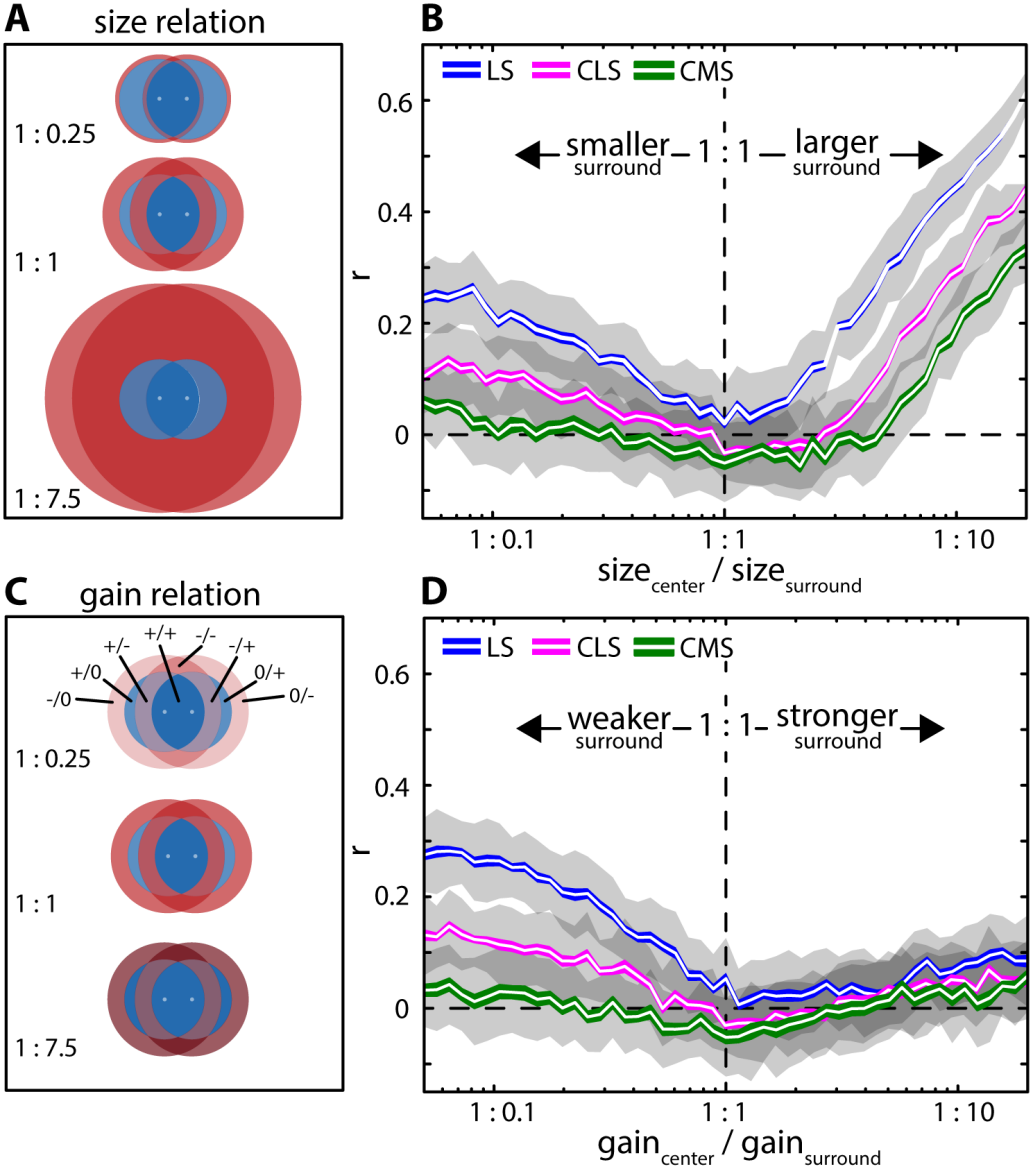
RF center-surround balance shapes correlations. **(A)** Model including an antagonistic center-surround structure which increased the complexity of the inputs (see Methods for detailed description). Shown are RF centers (blue) and surrounds (red) for three different RF center-surround size values (top: 1: 0.25; middle: 1: 1; bottom: 1: 7.5). In this plot, RF center size and overlap is that of LS as per anatomical data. **(B)** Correlation coefficients as a function of RF surround relative size for the three ELL maps (blue: LS; magenta: CLS; green: CMS). Differences between the three segments in terms of RF center size and % overlap were modeled according to available anatomical data (see Methods). Shown are the mean (white lines), SEM (colored areas) and STD (gray areas) of spike-count correlations calculated for t = 100 ms. **(C)** RF centers (blue) and surrounds (red) for three different RF surround gain values (top: 1: 0.25; middle: 1: 1; bottom: 1: 7.5). We separated the areas of RFs interactions into different sub-regions that were denoted by “+” if part of the center, “-”if part of the surround, and “0” if there is no input to a particular pyramidal cell. For example, “+/-”indicates the sub-region consisting of the overlap between the RF center of neuron 1 and the RF surround of neuron 2. **(D)** Correlation coefficients as a function of RF surround relative gain. Shown are the mean (white lines), SEM (solid areas) and STD (gray areas) for spike-count correlations calculated for t = 100 ms.

In order to explain the discrepancy between our model prediction and our experimental data, we refined our model to account for both RF center and surround. We then systematically varied RF surround size and gain relative to those of RF center to explore how RF center-surround organization influences correlations (Fig 4). This is because experimental data has shown large differences in RF surround size and strength between ELL maps [28].

Our results show that RF surround gain and size strongly influence correlated activity. First, consistent with previous results, correlation coefficient estimates obtained for small RF surround size tended towards those obtained in the absence of surround (compare Fig 4B with Fig 2). Increasing RF surround size decreased correlations for all three segments: estimates were lowest with RF center and surround size being equal. Further increasing surround size increased correlations for all three segments (Fig 4B). Qualitatively similar results were obtained when instead varying RF surround gain: increasing surround gain to a value similar to that of the RF center decreased correlations (Fig 4D). However, when further increasing RF surround gain, correlations increased much less than what observed when increasing surround size (compare Fig 4D & 4B). This is because the impact of the different areas of the RF interactions that determine correlations varies nonlinearly when RF surround size is changed, but linearly when RF surround gain is varied. Correlations varied most strongly for high RF center overlaps (i.e. LS) and less strongly for lower RF center overlaps (e.g. CLS & CMS). These results were qualitatively consistent across time scales (S7 Fig). Thus, our modeling predicts that the RF center-surround balance strongly influences correlations. Our model further predicts that correlations are more sensitive to changes in relative RF surround size than to changes in relative RF surround gain.

**Fig 5:**
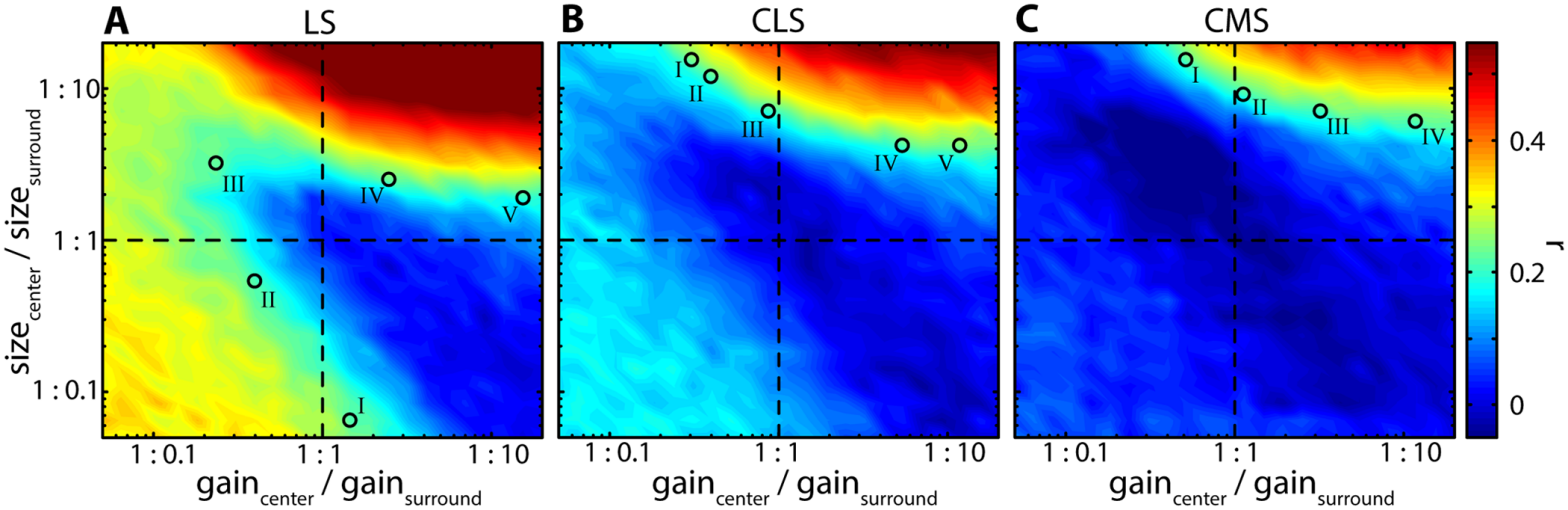
Different RF center-surround geometries give rise to similar correlations. **(A-C)** Spike-count correlations for t = 100 ms for LS (A), CLS (B), and CMS (C) as a function of RF surround relative gain (x-axis) and size (y-axis). At the intersection of the dashed horizontal and vertical lines RF center-surround balance is at equilibrium (i.e. equal gain and size between surround and center). Correlation magnitudes like those found experimentally (≈ 0.19; green color) were obtained for multiple and very different combinations of RF surround relative gain and size in all segments. Roman numerals (I—V) in each panel show example selected combinations of surround relative gain and size that were considered for further analyses.

To test whether different RF geometries can lead to similar levels of correlations, we next varied both RF surround size and gain jointly, while keeping the amount of RF center overlap at constant values corresponding to each of the ELL maps (Fig 5). In all three cases, we found that correlations were close to zero when RF center and surround were matched in strength (center of Fig 5A–5C; dark blue). Correlations increased strongly if the surround gain and size were increased (upper right quadrants in each plot) and less so if surround gain and size were decreased (lower left quadrants). If the surround size was increased while its gain was simultaneously decreased, we found that correlations on average increased in LS and CLS (Fig 5A & 5B; upper left quadrants). If the surround size was decreased while increasing its gain, correlations remained small in all cases (lower right quadrants). While the overall effects of RF surround size and gain on input correlations were qualitatively similar for all three segments, the ranges over which correlations varied differed. Specifically, for LS, we found the largest range of mean correlation coefficients (Fig 5A; ≈ -0.02–0.68). For CLS, we found a slightly smaller range (Fig 5B; ≈ -0.04–0.52). For CMS, we found the smallest range (Fig 5C; ≈ -0.07–0.45).

In general, for each segment, our model produced a large range of correlation values that were similar to the experimentally observed range (Fig 3). The values of RF surround gain and size for which correlations matched the average experimentally observed values (i.e., ≈ 0.19; blue to green color in Fig 5) however differed between segments. For LS (Fig 5A), physiological values of input correlations were obtained by manipulating center-surround balance in multiple ways. For example, by: strongly decreasing the surround size at a relatively balanced gain (e.g. “I” in Fig 5A); or by decreasing both surround size and gain (“II” Fig 5A); or by decreasing surround gain and increasing surround size (“III” in Fig 5A), or by increasing both surround size and gain (“IV” & “V” in Fig 5A). While similar results were obtained for CLS (Fig 5B) and CMS (Fig 5C), physiological levels of correlation were obtained only when strongly increasing surround size and either decreasing or increasing surround gain. Thus, our model predicts that physiologically realistic values of correlations can be obtained with different combinations of RF surround relative size and gain. Specifically, our model predicts that both weak and strong surround strength (i.e., in terms of both size and gain) can lead to equal levels of positive correlations.

We next determined the correlation transfer in our model: we found that output spike train correlations were effectively linearly scaled by a factor of ~ 0.49 with respect to the input correlations (i.e., the correlations between the inputs to both model ELL neurons, S8 Fig). We thus developed a reduced, theoretical model). In this model, the input to each neuron is described by the sum of all RF sub-portions which each are described by the product of its gain and its surface area. It is then possible to obtain an analytical expression for the correlation coefficient (see Methods). The output correlations were then obtained by scaling the input correlations by the factor found empirically in our numerical simulations. Results obtained using this reduced model were qualitatively similar to those obtained using the full model (compare S9 Fig to Fig 5). Thus, our results show that the effects of varying RF geometry on correlations in our full model can be, up to a scaling factor, explained by input correlations computed from the linearly summed inputs from different RF sub-regions.

**Fig 6:**
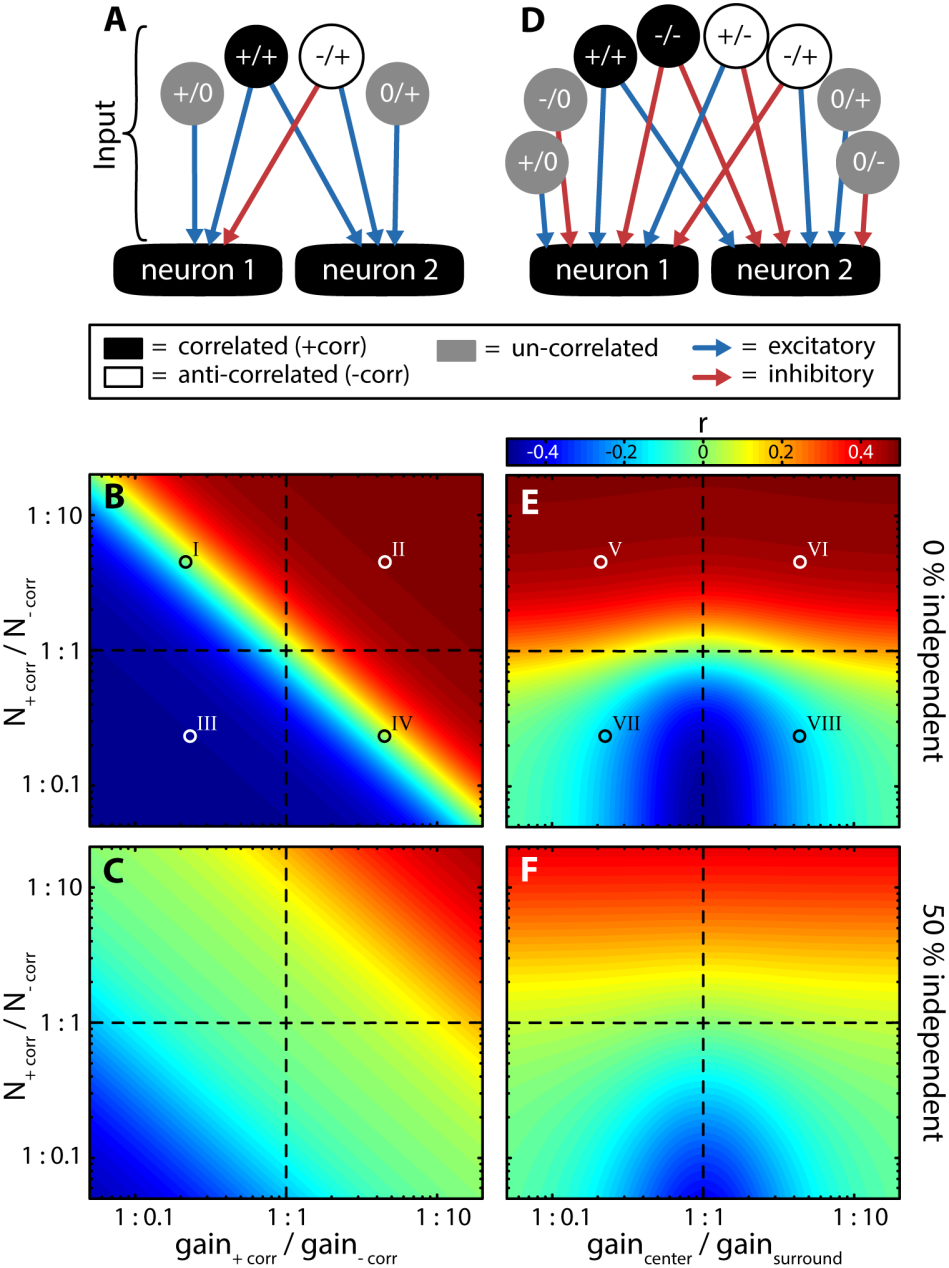
Effects of the different portions of RF interactions on correlated activity using arbitrary RF geometries. **(A)** Three types of inputs to two putative neurons were considered: (1) correlated input (black; same sign; +/+); (2) anti-correlated input (white; opposite sign; -/+); and (3) uncorrelated input (gray; +/0 & 0/+). The inputs were mapped onto the neurons either in an excitatory (blue arrows) or inhibitory (red arrow) fashion. **(B & C)** For a fixed total input (*N*_*tot*_ = 1000), the ratio of correlated and anti-correlated inputs was varied in terms of both their amount (*N*_*+corr*_ / *N*_*-corr*_) and in terms of their gain (*gain*_*+corr*_ / *gain*_*-corr*_). This was carried out for different but fixed portions of uncorrelated inputs (shown are 0% in B and 50% in C). Roman numerals (I–IV) depict cases for which the inputs are illustrated in S10A Fig **(D)** Expanded model in which all possible types of RF interactions were considered: correlated inputs (+/+ & -/-), anti-correlated inputs (+/- & -/+) and uncorrelated inputs (+/0, -/0, 0/+, & 0/-). These were mapped onto the downstream neurons either excitatory (blue arrows) or inhibitory (red arrows) depending on whether a given input was part of the RF center of the RF surround. **(E & F)** As (B & C), but when independently varying *gain*_*center*_ / *gain*_*surround*_ depending on whether an input was part of the RF center or surround. Roman numerals (V–VIII) depict cases for which the inputs are illustrated in S10B Fig.

In order to gain further understanding as to how interactions between different RF sub-regions determine output correlations, we considered a model with arbitrary RF geometry (Fig 6A, see Methods). Specifically, we segregated the inputs to both model neurons into those that are positively correlated (i.e., “+/+”, representing input coming from regions of overlap between both RF centers and overlaps of both surrounds), negatively correlated (i.e., “+/-“, representing input coming from regions of overlap between RF center with surround), and uncorrelated inputs (i.e., “+/0” and “0/+” representing regions for which there is no overlap between the RFs). This model allowed us to explore how arbitrary relations between these inputs shape correlations without having the constraints imposed by a particular RF structure (see Methods). We varied the ratio of correlated to anti-correlated input in terms of their amounts (i.e., “N”) and gain while keeping the total input fixed. In the absence of uncorrelated input (0% independent), the sign and magnitude of correlations was determined by the relative strength (i.e., that obtained when considering both amount and gain) of correlated and anti-correlated inputs (Fig 6B). Indeed, correlations were positive if the correlated input was dominant and negative if the anti-correlated input was dominant. Correlations were null if both correlated and anti-correlated inputs were matched in strength. Increasing the amount of uncorrelated inputs led to reduced correlations (i.e., “dilution”) but did not affect these results qualitatively (Fig 6C).

In the results shown in Fig 6B and 6C, we independently varied the gain of correlated, anti-correlated, and un-correlated inputs. This however, is physiologically not plausible. For example, the sign and gain of an input to a given cell depend only on whether this input is part of the RF center or surround region and not on the type of interaction with the RF of a neighbouring cell (i.e., both input sign and gain are “single-cell” properties). We thus considered an alternative model in which the gains and signs of the inputs to a given neuron are mapped as either being part of the RF center or RF surround, but where the amount of input originating from different regions of overlap between the RFs can be varied independently of one another (Fig 6D). For example, correlated inputs now originate both from regions of overlap between the RF centers (i.e., “+/+” in Fig 6D) and between the RF surrounds (i.e., “-/-”in Fig 6D). We then varied the ratio of correlated to anti-correlated inputs in terms of their amount while simultaneously varying the gain of the surround relative to that of the center. Our results are in agreement with those obtained using the reduced model in that the relative amount of positively correlated input determined the sign and magnitude of correlations and that addition of uncorrelated input effectively “diluted” correlations but otherwise did not affect results qualitatively (compare Fig 6E & 6F to Fig 6B & 6C, respectively). However, in contrast to the reduced model (Fig 6B & 6C), major differences were observed when varying the relative surround gain: similar levels of correlation were observed when the RF surround was either weak or strong (i.e., inverting the ratio of center/surround gain did not affect correlation magnitude. Fig 6E & 6F). This is because the net strength of positively correlated input relative to that of negatively correlated input are the same in both cases. It is important to realize however that the source of positively and negatively correlated inputs does vary. For example, the primary source of positively correlated input will come from RF center overlap in the case of weak surround and from RF surround overlap in the case of strong surround. Thus, these results explain why equal levels of correlation can be achieved with widely different RF structures as seen in our numerical simulations (Fig 5).

### Known differences in RF center and surround properties across the ELL maps lead to similar correlation magnitudes

**Fig 7:**
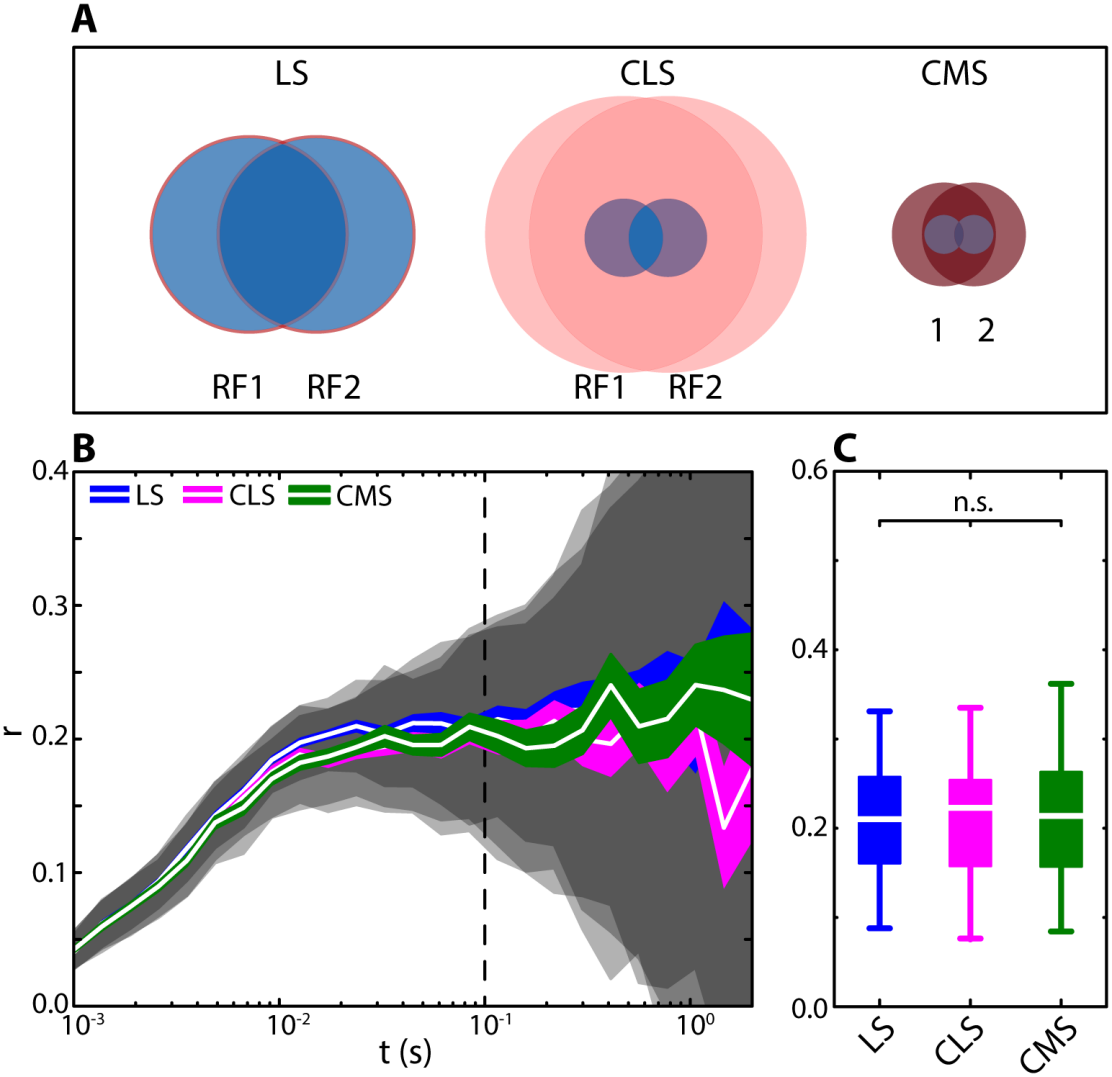
Experimentally measured differences in RF structure across the ELL maps give rise to similar levels of correlation in our model. **(A)** RF organization for our model LS, CLS and CMS neurons. These are based on previously published experimental data [28] and our numerical simulations. The impact of RF surround on correlations was small in LS (size relation = 1:0.065; gain relation = 1:1.47), moderate in CLS (size = 1:12; gain = 1:0.4), and strong in CMS (size = 1:6; gain = 1:12). **(B)** Spike-count correlations for LS (blue), CLS (magenta) and CMS (green) as a function of time window corresponding to the RF structures shown in (A). Shown are mean (white lines), SEM (colored areas) and STD (gray shaded areas) correlation coefficients calculated across 50 realizations of the model. The correlation coefficients predicted for the three segments were similar and thus largely overlapped with one another. (at T = 100 ms: median LS: 0.24, range: -0.44–0.79; CLS: 0.24, -0.57–0.67; CMS: 0.26, -0.20–0.71). **(C)** Distribution of correlation coefficients calculated for t = 100 ms (see vertical dotted line in B). At this timescale, the means of the distributions were not significantly different from one another (Kruskal-Wallis; dF = 2; Chi^2^ = 1.8; p = 0.41). Qualitatively similar results were obtained for other time windows (e.g.: t = 10 ms; Chi^2^ = 4.27; p = 0.12; or t = 1 s; Chi^2^ = 1.23; p = 0.54).

Finally, we tested whether including experimentally measured differences in RF structure across the three ELL maps [28,30] in our model can lead to equal average levels of correlations as found experimentally (Fig 3). The different RF structures are shown in Fig 7A. For LS, RF center size was greatest with large overlap with surround gain approximately to that of the center but with a very small relative size (Fig 7A, left; “I” in Fig 5A). For CLS, RF center size was smaller with a smaller amount of overlap while the surround size was larger with a very weak gain (Fig 7A, middle; “II” in Fig 5B). For CMS, RF center size was smallest with the lowest amount of overlap while RF surround size was large and RF surround gain was strong relative to that of center (Fig 7A, right; “III” in Fig 5C). We found that our model gave rise to correlations that were: 1) similar across all timescales despite these very different RF structures (Fig 7B & 7C) and; 2) similar to the average levels measured experimentally (compare Fig 7B & 7C with Fig 3B & 3C). Furthermore, the different RF structures across ELL maps led to physiologically realistic variations in correlation magnitude when the relative overlap between RF centers was varied (S11 Fig).

We conclude that the similar average levels of correlated activity displayed by ELL pyramidal cell pairs in the different ELL maps can be explained by considering differences in both RF center and surround specifically by the differing RF surrounds which influence correlation magnitude in each of the segments differentially. Indeed, including the RF surround led to a decrease in correlations for LS and an increase in correlations in CMS relative to values obtained when only considering the RF center (compare Fig 7 to Fig 2). For LS, our model predicts that correlations are primarily due to the strong positively correlated input originating from the large overlap between the RF centers. For CMS however, correlations are instead primarily due to the strong positively correlated input originating from the large overlap between the RF surrounds. In the case of CLS, the positively correlated input originating from regions of overlap between the RF centers and surrounds are somewhat attenuated by negatively correlated inputs originating from regions of overlap between the RF center and surround. We note that, within each segment, large heterogeneities in correlation magnitude were observed experimentally. These are likely due to heterogeneity in RF center overlap as well as differences in the relative size and gain of surrounds within each map as discussed below.

## Discussion

### Summary of results

We investigated the mechanisms that give rise to correlations between the baseline activities of ELL pyramidal neurons within three parallel somatotopic maps of the body surface. Using a simple feedforward model, we initially predicted that the decreasing amounts of RF center interactions from LS to CMS should lead to decreasing levels of correlated activity. In contrast, *in vivo* recordings revealed that the average levels of correlation between pyramidal neuron activities were nearly identical across all three segments. To explain this surprising result, we considered the effects caused by the antagonistic center-surround organization of RFs. By including and systematically varying the size and gain of a RF surround, we found that very different combinations of RF center-surround balance gave rise to nearly identical correlations between pyramidal neuron activities in our model. Further simulations showed that the sign and magnitude of correlations were largely determined by the relative strength of positively and negatively correlated inputs. However, it is impossible to vary the gains of these independently when considering a physiologically realistic RF center-surround organization. Taking this fact into account showed that, while the relative amount of positively and negatively correlated inputs still largely determined correlation sign and magnitude, relative gain was less important. This was because both a relatively weak or a strong surround gives rise to the same correlation magnitude. Finally, we showed that including the previously published differences in RF center-surround structure across the three ELL maps in our model resulted in physiologically realistic magnitudes of correlations that were similar across segments. Thus, our results strongly suggest that pyramidal cell pairs within different ELL segments display similar average levels of correlated activity because of known differences in RF center-surround organization.

### Assessing the impact of RF center-surround organization on correlated activity

Our results show that the RF structure has a strong influence on correlated activity between neurons. It should be noted that we assumed that RFs in our model were circular and organized in an antagonistic center-surround fashion. Experimental data has shown that ELL pyramidal neuron RF centers can be more elongated along the animal’s rostro-caudal axis [22,28]. This is not expected to alter the qualitative nature of our results, as increasing RF eccentricity will lead to relatively greater amounts of RF overlap, thereby scaling both positively and negatively correlated inputs similarly. We further note RF center and surround inputs to pyramidal neurons are excitatory and inhibitory, respectively. Thus, by altering the RF geometry, we effectively altered the balance of excitation and inhibition in the input to ELL neurons. Our modeling results thus agree with previous theoretical studies showing that balanced excitation and inhibition can cause a decorrelated state (i.e. correlations are negligible) [3,9].

The present study characterizes correlations between the baseline activities (i.e., in the absence of stimulation) of ELL pyramidal neurons. As mentioned above, baseline correlations are the limit towards which noise correlations will tend to as stimulus intensity decreases. Thus, for weak stimulus intensities, baseline and noise correlations will be approximately equal to one another (see Methods). Generally, noise correlations are thought to be caused by shared neuronal input and consequently, experimentally measured correlations were found to depend on the proximity of neurons in visual cortex [4,41] or on the amount of RF center overlap in the retina and the electrosensory system [7,12,42]. We here extend this notion to the RF surround as we have shown that it is the balance between the RF center and the RF surround that can have substantial impact onto determining correlations.

We note that the correlation magnitudes observed experimentally within each segment showed considerable heterogeneity. While these can partly be accounted to the varying degree of RF center overlap that RF will show within each segment [7], our model also predicts that differences in the relative surround size and gain will strongly affect correlation magnitude. While differences in size and gain have been observed experimentally [22,28], further studies are needed to verify whether and how these contribute to giving rise to differences in correlation magnitude in vivo.

There exists similarities between the electrosensory and other systems (see [17] for review). For example, RFs with an antagonistic center-surround organization are a common feature in the peripheral organization of sensory systems and are found in visual [27], somatosensory systems [43], in the lateral line system of fish [44], and in the auditory system [45]. Consequently, our results showing how differences in RF center-surround structure affect correlated activity are likely to be applicable to other systems. As discussed below, these correlations are expected to strongly affect neural coding.

### Role of correlated activity in information coding

The role of correlations in information coding has been extensively studied and is the focus on much debate (see [2] for review). On the one hand, correlations can be detrimental to coding because of redundancy. On the other hand, more recent studies have uncovered a more positive role for correlations [5,6]. Theoretically it was shown that, in order to assess the effect of noise correlations on coding, one must consider their sign with respect to that of signal correlations [2]. When noise and signal correlations have the same sign, noise correlations introduce additional variability compared to the independent case, and thus reduce coding efficacy. In contrast, when signal and noise correlations have opposite sign, the variability is effectively reduced thereby increasing coding efficacy. However, understanding how noise correlations impact the neural code in general is greatly complicated by the fact that they are plastic and can be modulated by several factors including stimulus properties as well as the animal’s behavioral state [1,4,7,8,46]. Recent studies have only begun to uncover the means by which state dependent correlations can aid in the decoding of behaviorally relevant stimuli [5,6].

### Role of correlations in electrosensory processing

Our recordings show that pyramidal cells display significant baseline correlations in all three ELL segments. This implies that pyramidal cells will display noise correlations under stimulation, specifically for low stimulus intensities. In fact, the presence of noise correlations under stimulation has previously been shown for CLS and LS neurons [7,8] and this is expected to impact the processing of behaviorally relevant electrosensory stimuli as discussed below.

Behavioral studies have shown that weakly electric display excellent (> 90%) accuracy at detecting electrical signals caused by prey [47,48]. At the time of detection, the perturbations of the animal’s own electric field caused by the prey are very weak and will activate only a portion of the animals’ sensory epithelium. This will in turn cause very small perturbations in the activities of afferents and ELL pyramidal neurons in all three ELL maps [16,49]. This is because their frequency tuning [24,28,50] and RF structure [30,49] are optimized to the resulting input statistics. Perturbations to the electric field caused by prey can in theory be accurately predicted by averaging the activities of large ELL pyramidal cell populations [31]. This assumes however, that noise correlations are negligible. Our results strongly suggest that this will not be the case for pyramidal cells in all maps including CMS. The impact of noise correlations on the detection performance of the ELL pyramidal cell population has not been investigated to date and should be included in future studies. In particular, the dependency of noise and signal correlations on the spatial position of a stimulus should be investigated systematically in order to determine the sign relationship between signal and noise correlations and thereby assess their impact on coding, similar to what has been done recently in other systems [5,6].

Beyond prey signals, ELL pyramidal neurons also respond to electro-communication stimuli arising during social interactions between conspecifics. When two fish come into proximity of one another (< 1 m), each fish will experience a sinusoidal amplitude modulation of its EOD (i.e., a beat). Natural electro-communication signals consisting of brief (< 30 ms) changes in EOD frequency and/or amplitude that will cause transient distortions in this beat signal [51]. While there has been much interest in understanding how electro-communication stimuli are encoded by single electrosensory neurons [52–57], only a few studies have investigated their encoding by neural populations [7,8,33]. Interestingly, these stimuli will activate most if not the entire animal’s sensory epithelium, and furthermore activate feedback inputs projecting to the ELL pyramidal cells from higher order brain areas [58,59]. Activation of feedback input has been shown to reduce noise correlations in CLS and LS neurons [7,8,33]. Nonetheless, the resulting “weak” correlation coefficients (~ 0.1) can still have strong effects on coding when large neural populations are considered [60]. Previous studies have shown that signal and noise correlations have the same sign when stimulated with electro-communication stimuli [7]. Based on theoretical arguments, it could thus be expected that the presence of noise correlations will have a negative impact on the encoding of these signals. However, in naturalistic behavior stimulus intensity will vary dynamically as both conspecifics move with respect to one another [61,62]. It is likely that these changes in stimulus intensity will alter the level of noise correlations dynamically [7,8], which could presumably optimize information processing through a stimulus dependent adaption of correlation levels and/or relations. Further, several recent studies have proposed mechanisms by which correlated activity from electrosensory neurons can be decoded. In particular, it was shown that downstream neurons respond to increases in correlated afferent neuron activity in order to give rise to perception [56,63], leading to the interesting hypothesis that correlated activity carries behaviorally relevant information. In conclusion, the role of correlations in electrosensory stimulus coding is likely to be complex and should be the focus of future studies.

## Material and methods

### Experimental procedures

#### Animals

Specimens of *Apteronotus leptorhynchus* were acquired from tropical fish dealers and housed in groups with up to 15 fish (water temperature 29 ± 2°C; water conductivity of 100–300 μS cm^-1^). Animals were fed with blood worms and brine shrimp to satiation 5 times a week and were kept according to published guidelines [64]. All procedures were approved by McGill University’s animal care committee.

#### Electrophysiology

Surgical procedures and recording methods have been described in detail before [7,22,56,65]. Animals (13 ± 2 cm; N = 29) were immobilized with an intramuscular injection of tubocurarine (200 μl injection; 2 mg ml^-1^) and transferred to the experimental tank (≈ 10 L). A constant water flow (≈ 10 ml min^-1^) over the gills was provided throughout surgery and experiments for aeration. After topical application of a local anesthetic (2% lidocaine, Western Medical Supply, Arcadia, CA, USA) a small craniotomy (≈ 5 mm^2^) was made in the dorsal skull to access the hindbrain. For recordings two metal-filled micropipettes [7,33] were inserted simultaneously into the pyramidal cell layer of the respective ELL segment. Segments were differentiated based on electrode angle and placement relative to the surface of the brain as well as by the monitored recording depth, similar to previous studies [24]. Recorded pairs were either registered on the same electrode (same-electrode pairs) or on separate electrodes (different-electrode pairs). As no significant differences were seen, data from same-electrode pairs and different-electrode pairs were pooled unless mentioned otherwise.

Electrode signals were amplified and filtered (x1000, 300–5 kHz; AM differential amplifier 1700; AM Systems, Sequim, WA, US) and digitized at 20 kHz (CED Power 1401, Cambridge Electronic Design, Cambridge, England). Recordings were stored for further analysis (Spike II, v7.16 x86, CED). Recording length ranged from 39 to 318 s (mean 105 s) and we only considered neural activities that were stationary during the recording.

#### Data analysis

For offline analysis, spikes were separated from the recorded traces using a threshold and assigned to different neurons based on waveform, inter-spike-intervals, and PCA with subsequent k-means or normal-mixtures clustering (Spike II, v7.16 x86, CED). All further analyses and modelling procedures were carried out using customized routines in Matlab-software (MATLAB R2015b v8.6.0, MathWorks Inc., Natick, MA, US).

In order to determine pyramidal cell type (i.e. ON- vs OFF-type), the animal was stimulated with a global amplitude modulation (random AM 0–120 Hz) of the EOD as described previously [7,56,66]. Type was determined through computation of a spike-triggered average (width 250 ms) as done previously [67,68]: ON-type neurons showed a prominent up-stroke prior to time 0 while OFF-type neurons showed a down-stroke, respectively.

The time series of each pair of simultaneous recorded spike trains was compared using spike count correlations at different timescales. For this, each spike train was converted to a time series of spike counts by summing the number of spikes that fell within a certain interval (spike count window) with given duration *t*. *t* was systematically varied in the range 10^−3^–2 s to obtain a correlation coefficient at the respective timescale. From the time series of spike counts the correlation coefficient *r* was calculated according the following equation:

$$r=\frac{Cov\left(n_{1}n_{2}\right)}{Var\left(n_{1}\right)\;Var\left(n_{2}\right)}$$ (1)

Where *n*_*i*_ is the spike count series obtained for neuron *i* for a given spike count window duration *t*, *Cov*(…) is the co-variance, and *Var*(…) is the variance [3,11,33,69]. The correlation coefficients represent baseline correlations (i.e. without stimulation) between the spike time series of two pyramidal neurons.

To obtain correlations magnitude for a given population, we averaged correlation coefficients as determined from neuron pairs. We note that this could, in principle, lead to improper estimates since the data from each pair are not necessarily independent from one another [70]. To test this, we compared correlation coefficients estimated from our entire dataset to those obtained from randomly chosen subsamples consisting of neuron pair recorded in different animals. We found that our correlation estimates obtained using the entire dataset was well within the distribution of correlation coefficients computed using these subsamples (see S1 Fig).

Time varying correlation coefficients were obtained using techniques similar to those described previously [71,72]. Spike counts were obtained for t = 100 ms and an evaluation window of 30 s width was slid across the data in steps of 100 ms. For each step, a correlation coefficient was obtained for the data within the evaluation window using Eq 1. The obtained time varying correlation coefficients did not vary substantially (S3A Fig).

Alternatively, correlation coefficients were obtained from the coherence function using $r=\sqrt{C\left(0\right)}$. Where the coherence function is given by:

C(f)=|P12(f)|2P11(f)P22(f). (2)

Here *P*_12_(*f*), is the cross-spectrum between the spiking responses of neurons 1 and 2, and *P*_*ii*_(*f*) is the power-spectrum of neuron *i*. We overall found good agreement between estimates of *r* obtained using Eqs 1 and 2 (S12 Fig).

#### Relationship between baseline and noise correlations

Let us consider two spike trains, X_1_(t) and X_2_(t) that receive a common stimulus *S(t)*. According to linear response theory [73], we have:

$${\tilde{X}}_{i}\left(f\right)={\tilde{X}}_{0i}\left(f\right)+{\tilde{X}}_{i}\left(f\right)\tilde{S}\left(f\right)$$ (3)

where *i* = 1,2; ${\tilde{X}}_{i}\left(f\right)$ is the Fourier transform of *X*_*i*_*(t);* $\tilde{S}\left(f\right)$ is the Fourier transform of *S(t);* ${\tilde{X}}_{0i}\left(f\right)$ is the Fourier transform of *X*_*0i*_*(t)* (i.e., the baseline activity in the absence of stimulation), and ${\tilde{X}}_{i}\left(f\right)$ is the susceptibility function. Linear response theory has been successfully applied to experimental data [35,74,75] in order to compute approximate expressions for neural responses, including correlations between neural spike trains, provided that the stimulus input is weak enough [71,72]. It should be noted that linear response theory tends to be most applicable in the case where there is spontaneous neural activity with large variability in the absence of stimulation, which is the case for ELL pyramidal cells [76].

Here we claim that, when the stimulus intensity is weak enough, the noise correlation coefficient will be equal to the correlation coefficient between the baseline activities. We start from the definition of the correlation coefficient (Eq 1) and use the following [77]:

$$r=\frac{\int_{-t}^{t}C_{12}\left(t^{\prime }\right)\frac{t-\left|t^{\prime }\right|}{t}dt^{\prime }}{\sqrt{\int_{-t}^{t}C_{11}\left(t^{\prime }\right)\frac{t-\left|t^{\prime }\right|}{t}dt}\sqrt{\int_{-t}^{t}C_{22}\left(t^{\prime }\right)\frac{t-\left|t^{\prime }\right|}{t}dt^{\prime }}},$$ (4)

where *C*_*ij*_*(t)* is the Fourier transform of *P*_*ij*_*(f)* (e.g., C_12_(t) is the cross-correlation function). Using the Wiener-Khintchine theorem, we get:

$$r=\frac{\int_{-\infty }^{+\infty }P_{12}\left(f\right)K_{t}\left(f\right)df}{\sqrt{\int_{-\infty }^{+\infty }P_{11}\left(f\right)K_{t}\left(f\right)df}\sqrt{\int_{-\infty }^{+\infty }P_{22}\left(f\right)K_{t}\left(f\right)df}},$$ (5)

where

$$K_{t}\left(f\right)\propto \frac{{\text{sin}}^{2}\left(\pi ft\right)}{{\left(\pi ft\right)}^{2}}.$$ (6)

We note that, when t→∞, we have *K*_*t*_(*f*)→*δ*(*f*). Updating Eq (5) then shows that the correlation coefficient r is then equal to the square root of the coherence function at zero frequency. Using Eq 3, we get the following expressions for the cross and power-spectra:

$$P_{12}\left(f\right)\approx P_{0,12}\left(f\right)+{\tilde{\chi }}_{1}^{*}\left(f\right){\tilde{\chi }}_{2}\left(f\right)P_{S}\left(f\right)$$ (7)

$$P_{ii}\left(f\right)\approx P_{0,ii}\left(f\right)+{\left|{\tilde{\chi }}_{i}\left(f\right)\right|}^{2}P_{S}\left(f\right)$$ (8)

where we have $P_{0,12}\left(f\right)=\;\langle {\tilde{X}}_{01}^{*}\left(f\right){\tilde{X}}_{02}\left(f\right)\rangle$ is the cross-spectrum of the baseline activities (i.e., the “baseline cross-spectrum”) and, similarly, $P_{0,ii}\left(f\right)=\;\langle {\tilde{X}}_{0i}^{*}\left(f\right){\tilde{X}}_{0i}\left(f\right)\rangle$ are the power spectra of the baseline activities (i.e., the “baseline power spectra”). Now, the noise correlation coefficient is given by:

$$r_{noise}=\frac{\int_{-\infty }^{+\infty }\left[P_{12}\left(f\right)-{\langle P_{12}\left(f\right)\rangle }_{S}\right]K_{t}\left(f\right)df}{\sqrt{\int_{-\infty }^{+\infty }\left[P_{11}\left(f\right)-{\langle P_{11}\left(f\right)\rangle }_{S}\right]K_{t}\left(f\right)df}\sqrt{\int_{-\infty }^{+\infty }\left[P_{22}\left(f\right)-{\langle P_{22}\left(f\right)\rangle }_{S}\right]K_{t}\left(f\right)df}},$$ (9)

where 〈*P*_*ij*_(*f*)〉_*S*_ is the expectation of *P*_*ij*_(*f*) with respect to the stimulus S [69]. Inspection of Eqs 7 & 8 shows that the only the rightmost terms depend on the stimulus since, by definition, the baseline activity is not correlated with the stimulus. Thus, we have *P*_*ij*_(*f*) − 〈*P*_*ij*_(*f*)〉_*S*_ = *P*_0,*ij*_(*f*). Eq 9 thus becomes:

$$r_{noise}=\frac{\int_{-\infty }^{+\infty }P_{0,12}\left(f\right)K_{t}\left(f\right)df}{\sqrt{\int_{-\infty }^{+\infty }P_{0,11}\left(f\right)K_{t}\left(f\right)df}\sqrt{\int_{-\infty }^{+\infty }P_{0,22}\left(f\right)K_{t}\left(f\right)df}}.$$ (10)

Thus, the noise correlation coefficient only depends on the baseline activities if the stimulus intensity is weak enough and is furthermore equal to the correlation coefficient obtained from the baseline activities, which was our original claim.

### Modelling correlated activity in ELL

Our model consists of two ELL pyramidal neurons each receiving input from a pool of receptor afferents. We note that this input could be from either feedforward or feedback sources as anatomical studies have shown that the feedback input received by a given pyramidal cell originates from a separate group of pyramidal cells [58]. Thus, any feedback input can effectively be considered a delayed feedforward input [33]. We further note that transmission delays are irrelevant here because on the one hand we are only considering baseline conditions (i.e., in the absence of stimulation), and on the other hand experiments have shown that the baseline activities of receptor afferents are not correlated with one another [35,56,72]. In general, model parameters were constrained based on available experimental data (see below) such as to fit average values unless otherwise noted.

#### Model of peripheral receptor afferents

To estimate the amount of correlations in the inputs to the model ELL neurons we calculated the correlations between the summed activity of simulated p-units that were combined according to the geometrical RF interactions. All modeling procedures were carried out using Matlab-software (MATLAB R2015b v8.6.0, MathWorks Inc., Natick, MA, US).

We simulated a pool (N = 13000) of electroreceptor afferent activities (p-units, 20 s) using a leaky integrate-and-fire model with dynamic threshold (LIFDT) that has been previously shown to accurately reproduce experimental results [36,37]. The model is described as follows:

$$\dot{V}=-\frac{V}{\tau_{V}}+\frac{I\left(t^{\prime }\right)}{\tau_{V}}$$ (11)

$$\dot{\theta }=\;\frac{\theta_{0}-\theta }{\tau_{\theta }}$$ (12)

$$I\left(t^{\prime }\right)=A_{0}+\sigma \;\xi \left(t^{\prime }\right)$$ (13)

Where *V* is the membrane potential, *τ*_*V*_ is the voltage decay constant of the membrane, *θ* is the spike threshold, *t*′ is time, and *τ*_*θ*_ is the threshold time constant. Whenever *V* reaches *θ* this results in a spike and *V* is set back to zero and is maintained there for the duration of the refractory period (*T*_*r*_). The threshold *θ* is also increased by a fixed amount *Δθ* from where it decays with *τ*_*θ*_. *I*(*t*′) is the total input current, which consists of a constant *A*_0_ and a Gaussian white noise process *σξ*(*t*′) with mean 0 and standard deviation *σ*. Thus, all neurons receive a constant offset input, while the independent Gaussian white noise process is meant to mimic sources of intrinsic variability (e.g., random channel opening and failures in synaptic transmission) to reproduce the experimentally observed variability in the spiking activity of afferents in the absence of stimulation (i.e., there is no stimulus driving the population). The model was simulated numerically using an Euler-Maruyama integration algorithm with time step 0.025 ms. Parameter values used were *τ*_*V*_ = 1 ms; *τ*_*θ*_ = 7.75 ms; *T*_*r*_ = 1 ms;*σ* = 12.65; *θ*_0_ = 0.08; *Δθ* = 0.05. To reproduce known heterogeneities in peripheral afferent baseline firing statistics [38,56,72], the parameter *A*_0_ was drawn from a Gaussian distribution with mean 1.7 and standard deviation 1. The model afferent population displayed a firing rate of 364 ± 90 Hz (mean ± STD), with a coefficient of variation of 0.194 ± 0.07 and probability of firing of 0.36 ± 0.09, all of which were in good agreement with experimental observations [38,56,72]. Afferent activities in the absence of stimulation were not correlated with one another, consistent with experimental results [35,56,72].

#### Modeling ELL pyramidal neurons RF center-surround interactions

Spiking activities of model afferents were summed to estimate the integrated input a pyramidal neuron receives from the afferent population. The number of model afferents used was set according to the geometrical estimation of the RF interaction between ELL pyramidal neurons (Fig 2A). For simplicity, we assumed that electroreceptors were uniformly distributed on the fishes’ skin and that RF center shape was circular. Size and overlap of the RF centers were fixed and set to average values determined by available anatomical data [30]. To simulate the differences in RF center size, we varied the number of model afferents (LS/CLS/CMS: 640/105/25) that were summed for a RF center. This is because anatomical measurements have shown that LS cells receive input from a large (600–1400), CLS from intermediate numbers (104–236), while CMS cells instead receive input from a small number (24–56) of afferents [30]. To simulate the differences in RF overlap (56.9/33.3/13.2%) we used a fixed number of common afferents (358/35/3) between both pyramidal cell models. We note that anatomical studies have shown that the amount of shared afferent input varies both between and within the maps (LS: 49.4–64.5%; CLS: 30.4–36.3%; CMS: 10.6–15.7%) [30]. We assumed that the center and surround gains were spatially uniform with a sharp transition at the border between the two, consistent with available experimental data [22].

In the case of RF center alone (i.e., no surround), input quantities were based on regions of overlap and non-overlap between two circles with same radius but whose centers were offset relative to one another. This resulted in three input categories (RF1/RF2: 0/+, +/+ and +/0) each consisting of a given number of afferents proportional to area.

When including the effects of RF surround, we added an annulus surrounding each RF center. We then considered all possible interactions between the resulting overlapping RFs which were defined by the different regions of overlap and non-overlap between the two circles (i.e., the centers) and annuli (i.e., the surrounds). That resulted in up to eight input categories (RF1/RF2: -/0, +/0, -/+, +/+, -/-, +/-, 0/+ and 0/-; see Fig 4C). The number of afferents corresponding to each region were set proportional to area as extrapolated using basic geometry. The activities were multiplied by -1 if originating from surround to account for surround antagonism and then summed. The resultant signals were then normalized and low-pass filtered (1^st^ order Butterworth filter at 50 Hz) to mimic synaptic filtering [78] before being used as input *I*_*aff*_(t′) to an ELL pyramidal neuron that was modeled using the leaky integrate-and-fire formalism:

$$C\;\dot{V}=-G_{leak}\left(V-E_{leak}\right)+I_{bias}+I_{aff}\left(t^{\prime }\right)$$ (14)

Where *V* is the membrane potential, *E*_*leak*_ = −70 *mV* is the reversal potential, *G*_*leak*_ = 0.36 μS is the leak conductance, *I*_*bias*_ is the bias current, and *C* = 1 *nF* is the membrane capacitance. Whenever *V* reaches the threshold *θ* = −35 *mV*, an action potential is said to have occurred and *V* is immediately reset to −70 *mV* where it is maintained for the duration of the refractory period *T*_*r*_ = 10 *ms*. Parameter values were chosen based on available experimental data [65]. The value of *I*_*bias*_ was adjusted for each simulation to set the output firing rate of the simulated neurons to 16.0 ± 0.5 Hz because experimental investigations have found that ELL pyramidal neurons in all three maps display average firing rates near 16 Hz in the absence of stimulation [24]. *I*_*bias*_ values used in our model were typically between -5.73 nA and 5.62 nA. The model was simulated numerically using an Euler-Maruyama integration algorithm with an integration time step of 0.025 ms.

Correlations were calculated for the output spiking activities of two simulated pyramidal neurons according to Eq 1. Input correlations were also calculated from the summed input spike trains to the model ELL neurons at different time windows according to Eq 1.

#### Changes in RF center-surround balance, center overlap

To change gain and size relations of RF center and surround, we always changed the properties of the RF surround while leaving those of the RF center constant. Both size and gain relation were varied along a logarithmic scale from 1:0.1 to 1:10 (center: surround). To change the gain of the surround, the surround signal was multiplied by a gain factor ranging from 0.1 to 10 while that of the center was kept at 1. To change the size relation, we scaled the surface area of the RF surround annulus by factors ranging between 0.1 and 10. We note that the correlation coefficient only depends on the ratio of center/surround gain and area (see below).

To change RF center overlap, the gain and size relation between center and surround were set to fixed values. After that, the distance between the RFs circles was adjusted to achieve the desired overlap of RF centers. In repetitive trials, the overlap was varied with a constant step size from 1% to 99%. For each respective overlap value, the p-unit quantities were extrapolated from the areas of the RF surface interactions and the respective numbers of p-units were multiplied and summed according to the methodology described above. All model data presented was generated based on at least 25 realizations of the model for which the composition of the input quantities (i.e. which simulated p-units were picked) was random.

### Theory of ELL correlations

Based on the different regions of overlap between the RFs (see above), the level of correlation arising from the inputs to both model pyramidal neurons can be computed analytically. For the example of two interacting excitatory RF centers with inhibitory surrounds the inputs for each of the pyramidal neurons *n*_*1*_ and *n*_*2*_ can be expressed as:

$$n_{1}=\left[\left(G_{c}N_{+/+}\right)+\left(G_{c}N_{+/-}\right)+\left(G_{c}N_{+/0}\right)-\left(G_{s}N_{-/-}\right)-\left(G_{s}N_{-/+}\right)-\left(G_{s}N_{-/0}\right)\right]$$ (15)

$$n_{2}=\left[\left(G_{c}N_{+/+}\right)+\left(G_{c}N_{-/+}\right)+\left(G_{c}N_{0/+}\right)-\left(G_{s}N_{-/-}\right)-\left(G_{s}N_{+/-}\right)-\left(G_{s}N_{0/-}\right)\right]$$ (16)

Where N is the number of afferents according to the size of a given area of interaction and G is the gain of the respective area, with G_c_ used if the area is part of the RF center and G_s_ if the area is part of the RF surround. Note that the areas are computed using Euclidian geometries based on the intersection between the antagonistic RF structures. The % overlap was varied by changing the distance between the two centers of the circles representing the RF centers. The variances of the inputs are then given by:

$$Var\left(n_{1}\right)=G_{S}^{2}\left[{\left(\frac{G_{c}}{G_{s}}N_{+/+}\right)}^{2}+{\left(\frac{G_{c}}{G_{s}}N_{+/-}\right)}^{2}+{\left(\frac{G_{c}}{G_{s}}N_{+/0}\right)}^{2}+{\left(N_{-/-}\right)}^{2}+{\left(N_{-/+}\right)}^{2}+{\left(N_{-/0}\right)}^{2}\right]$$ (17)

$$Var\left(n_{2}\right)=G_{S}^{2}\left[{\left(\frac{G_{c}}{G_{s}}N_{+/+}\right)}^{2}+{\left(\frac{G_{c}}{G_{s}}N_{-/+}\right)}^{2}+{\left(\frac{G_{c}}{G_{s}}N_{0/+}\right)}^{2}+{\left(N_{-/-}\right)}^{2}+{\left(N_{+/-}\right)}^{2}+{\left(N_{0/-}\right)}^{2}\right]$$ (18)

The covariance of the signals is then given by:

$$Cov\left(n_{1},n_{2}\right)=G_{s}^{2}\left[{\left(\frac{G_{C}}{G_{S}}N_{+/+}\right)}^{2}+{\left(N_{-/-}\right)}^{2}-{\left(\frac{G_{c}}{G_{s}}N_{+/-}\right)}^{2}-{\left(\frac{G_{c}}{G_{s}}N_{-/+}\right)}^{2}\right]$$ (19)

Using the variance and the covariance we calculated the correlation according to:

$$r=k\;\cdot \frac{Cov\left(n_{1},n_{2}\right)}{\sqrt{Var\left(n_{1}\right)\;Var\left(n_{2}\right)}}$$ (20)

Where *k* is a constant accounting for the correlation transfer from the input to the output stage of the ELL as determined from our LIF model (see S8 Fig). We empirically used *k* = 0.4884 based on our numerical simulations. Inspection of Eqs 8–11 makes clear that, while both variance and covariance explicitly depend on *G*_*s*_ and *G*_*c*_ (Eqs 17–19), the correlation coefficient *r* (Eq 20) only depends on the ratio of *G*_*s*_ and *G*_*c*_.

In order to calculate correlations for the interactions of RFs with arbitrary geometry, we adjusted our mathematical model accordingly. For the calculations shown in Fig 6A–6C, inputs were used according to:

$$n_{1}=\left[\left(G_{+corr}\;N_{+corr}\right)-\left(G_{-corr}\;N_{-corr}\right)+\left(N_{01}\right)\right]$$ (21)

$$n_{2}=\left[\left(G_{+corr}\;N_{+corr}\right)+\left(G_{-corr}\;N_{-corr}\right)+\left(N_{02}\right)\right]$$ (22)

With *G*_*+corr*_ and *N*_*+corr*_ representing the gain and amount of correlated input, *G*_*-corr*_ and *N*_*-corr*_ represent the gain and amount of anti-correlated input, respectively. *N*_*01*_, *N*_*02*_ are the amounts of uncorrelated inputs to each neuron *n*_*i*_.

The variances of inputs *n*_*1*_ and *n*_*2*_ were calculated using:

$$Var\left(n_{1}\right)=\left[{\left(G_{+corr}\;N_{+corr}\right)}^{2}-{\left(G_{-corr}\;N_{-corr}\right)}^{2}+{\left(N_{01}\right)}^{2}\right]$$ (23)

$$Var\left(n_{2}\right)=\left[{\left(G_{+corr}N_{+corr}\right)}^{2}+{\left(G_{-corr}N_{-corr}\right)}^{2}+{\left(N_{02}\right)}^{2}\right]$$ (24)

Covariance was calculated using:

$$Cov\left(n_{1},n_{2}\right)={\left(G_{+corr}N_{+corr}\right)}^{2}-{\left(G_{-corr}N_{-corr}\right)}^{2}$$ (25)

The correlation coefficient was then computed according to Eq 20. We note that the parameters G and N are completely interchangeable and thus will have similar effects on correlations in this reduced version of the model. Further, in our investigations, we kept the total amount of input constant (i.e., *N*_*tot*_ = *N*_*+corr*_ + *N*_*-corr*_ + *N*_*01*_ + *N*_*02*_ = 1000) and assumed that *N*_*01*_ = *N*_*02*_. Changing the total amount of input had no effect on the shown characteristics of *r*.

For the calculations in Fig 6D–6F, we used the original mathematical model (Eqs 15–20) and split the areas of correlated signals equally between the terms A_+/+_ and A_-/-_, areas of anti-correlated inputs between A_+/-_ and A_-/+_, and uncorrelated input between A_+/0_, A_-/0_, A_0/+_ and A_0/-_

## Supporting information

S1 Fig Averaging correlation coefficients leads to accurate estimates of correlation magnitude. **(A)** Population-averaged absolute correlation coefficient for our entire CLS dataset (black line; N = 108) and for different sub-populations (gray lines; 20 random examples shown; each N = 17) generated by randomly selecting neuron pairs recorded in different animals. **(B)** Distribution of correlation coefficients obtained from independent sub-populations (gray area generated from 10^10^ different subpopulations) for a timescale of 100 ms (see vertical dotted line in (A)). The correlation coefficient obtained from the entire dataset (horizontal dotted line) was located within 42% of the area und the curve of the distribution, which is well below 95%.
(TIF)

S2 Fig Correlations do not depend on recording technique. **(A)** Population-averaged absolute correlation coefficient for CLS pairs recorded on the same electrode (N = 26) as a function of time window. **(B)** Same as A, but for CLS pyramidal cell pairs that were recorded on separate electrodes (N = 82). In (A) and (B), shown are the mean (white line), SEM (black area) and STD (gray areas) of the population of pairs. **(C–E)** Population-averaged absolute correlation coefficients for same-electrode (“same”) and different-electrode (“different”) pairs in CLS at time windows (T) of 15 ms (C), 100 ms (D) and 1 s (E, see vertical dotted line in A & B). For each time window, the population-averaged absolute correlation coefficients obtained for same-electrode and different-electrode pairs were not significantly different from one another (Kruskal-Wallis: df = 1; Chi^2^ = 0/1.26/0.69/0.92; p = 0.95/0.26/0.34 for C/D/E).
(TIF)

S3 Fig Time varying correlation coefficients showed low variance for a given cell pair. **(A)** Time varying spike count correlation coefficients (t = 100 ms, see Methods) from five example pairs. **(B)** Distributions of time varying correlation coefficients for the same five example pairs were generally unimodal. **(C-E)** Variance of the time varying correlation coefficients for all pairs in LS (C), CLS (D) and CMS (E). Variances of time varying correlation coefficients were generally small (mean for LS = 4.2 · 10^−3^; CLS = 3.8 · 10^−3^; CMS = 3.7 · 10^−3^) and lower than the variance of correlation coefficients across the population (LS = 3.4 · 10^−2^; CLS = 5.3 · 10^−2^; CMS = 5.6 · 10^−2^) by an order of magnitude.
(TIF)

S4 Fig Correlation strength was independent of firing rate differences within pairs in CLS. **(A)** Correlation coefficients (t = 100 ms) as a function of the difference in firing rate between neurons in each pair. **(B-C)** Pooling the data depending of different ranges of firing rate differences (see horizontal lines in A) showed similar means and variability in all cases (B: Δ firing rate 0–3 Hz; C: 3–7 Hz; D: >7 Hz). The means of the data were not significantly different when compared at various timescales (Kruskal-Wallis, t = 100 ms: df = 2; Chi^2^ = 3.88; p = 0.14; t = 1 s: df = 2; Chi^2^ = 0.49, p = 0.78).
(TIF)

S5 Fig Correlation strength was independent of pair composition in CLS. **(A)** Spike count correlations as a function of time window for opposite type (ON-OFF) pyramidal cell pairs (N = 48). **(B & C)** Same as (A), but for ON-ON (N = 20) and OFF-OFF (N = 22) pairs, respectively. In all panels, individual lines depict correlations of individual pairs, with the color showing the geometric mean of the firing rates for each cell in the pair. The black lines are the population averages.
(TIF)

S6 Fig Correlations were firing rate dependent on small timescales in CLS. **(A–C)** Absolute correlation coefficient for individual pairs as a function of the geometric mean of the firing rates of each cell in the pair (black dots) for time windows of 5 ms (A), 25 ms (B), and 100 ms (C). Also shown are the best-fit straight lines. **(D)** Best-fit straight lines for different time window lengths (colored lines). The slope of the best-fit straight line first increased for time windows up to 25 ms and then decreased. **(E)** Goodness-of-fit R^2^ (triangles) and slope (circles) of the best-fit straight line as a function of time window.
(TIF)

S7 Fig Correlation coefficients obtained for different RF center-surround organizations are largely independent of timescale. **(A)** RF centers (blue) and surrounds (red) for three different RF center-surround size values (1:0.25, top; 1:1 middle; 1:7.5 bottom). RF center overlap is that of CLS as per anatomical data, gain relation of center to surround was fixed at 1:1. **(B)** Correlation coefficient as a function of timescale for different relative surround size values (see color-code). **(C)** Same as (A), but when instead varying RF surround relative gain. **(D)** Same as (B), but for different relative surround gain values. **(E)** Same as (A), but sketched for different RF center overlaps. **(F)** Same as (B), but for different RF overlap values. Surround relative gain and size were 0.4 and 11.88, respectively.
(TIF)

S8 Fig The spiking nonlinearity scales output correlations relative to input correlations. **(A)** Linear regressions obtained by linear least squares fit of output correlations as a function of input correlations for simulations mimicking the 3 segments (blue: LS; magenta: CLS; green: CMS). Shaded-areas represent one standard deviation of the data. Inset: Data from our simulations showing output as a function of input correlations. Our simulation results were similar to what was reported in earlier studies [11,71]. Importantly, the relationship between output and input correlation was linear in the range of physiologically observed correlation magnitudes (white area) based on our experimental data. **(B)** We fitted a linear regression on the pooled data from all segments in order to empirically obtain the scaling factor relating input to output correlations. We found k = 0.4884.
(TIF)

S9 Fig Theory showing how linear interactions between different regions of RF overlap of two neurons determine output correlations up to a constant scaling factor. **(A-C)** Correlations obtained from our mathematical scale model (Eqs 15–20) for LS (A), CLS (B) and CMS (C) while varying RF balance in terms of RF center to surround gain relation and RF center to surround size relation. The results from our mathematical model were in good quantitative agreement with our numerical simulations (compare to Fig 5).
(TIF)

S10 Fig Inputs in our theoretical model with arbitrary RF geometry. **(A—D)** In our theoretical model the relative amount of correlated (+/+) and anti-correlated (-/+) inputs was systematically varied in our model alongside the relative gain with which these inputs were mapped onto each respective neuron. Four (I–IV) or these input relations are visualized in the panel corresponding to the positions in Fig 6B. For each of the panels the left box visualizes how inputs are mapped onto neuron 1, while the right box visualizes the same inputs and how these are mapped onto neuron 2 (compare Fig 6A). Excitatory inputs are visualized in blue, inhibitory inputs in red, the saturation visualizes the strength of the gain. While the total amount of inputs was constant in all cases (A–D, total size of boxes) the relative amounts of input (horizontal divisions) was changed. **(E- F)** Same as (A-B) but for the model in which gains were mapped differentially according to the center vs. surround portion to which inputs belonged (compare Fig 6D). The panels correspond to the input relations (V–VIII) marked in Fig 6E.
(TIF)

S11 Fig Impact of changes in RF center overlap on correlations for different RF center-surround balances. **(A)** Correlation coefficient as a function of RF center overlap (in percent) using different values of relative surround gain and size corresponding to the points I-V shown in Fig 5A. Shown are the mean (white line), SEM (black area) and STD (gray area). We used RF center values as per anatomical knowledge form LS. The gray shaded area marks RF center overlap not found anatomically and thus divides the data into physiologically realistic (white) and physiologically non-realistic (shaded gray) values of RF center overlap. **(B)** Same as (A) but magnified such as to better highlight the differences between the curves within the physiological relevant overlap range. **(C & D)** Same as (A & B), but for CLS RF center size values. The red line and shaded area depicts the linear fit of physiological CLS data previously published in [7]. **(E & F)** Same as (A & B), but for CMS RF center size values.
(TIF)

S12 Fig Correlation estimates between spike counts at large time scales are in good agreement with those estimated from the coherence between spike trains. For all experimental CLS data we estimated correlation magnitude by taking the square root of the coherence between spike trains evaluated at frequency 0. There was good agreement between correlation estimates from the coherence and absolute correlation estimates from the spike count (red line is the best-fit straight line; R^2^ = 0.78; the dotted line shows the identity line).
(TIF)
