# Supplementary figures and images for: Differential receptive field organizations give rise to nearly identical neural correlations across three parallel sensory maps in weakly electric fish
Source: PLoS Comput Biol. 2017 Sep 1;13(9):e1005716. doi: 10.1371/journal.pcbi.1005716 (PMC5599069; doi:10.1371/journal.pcbi.1005716)

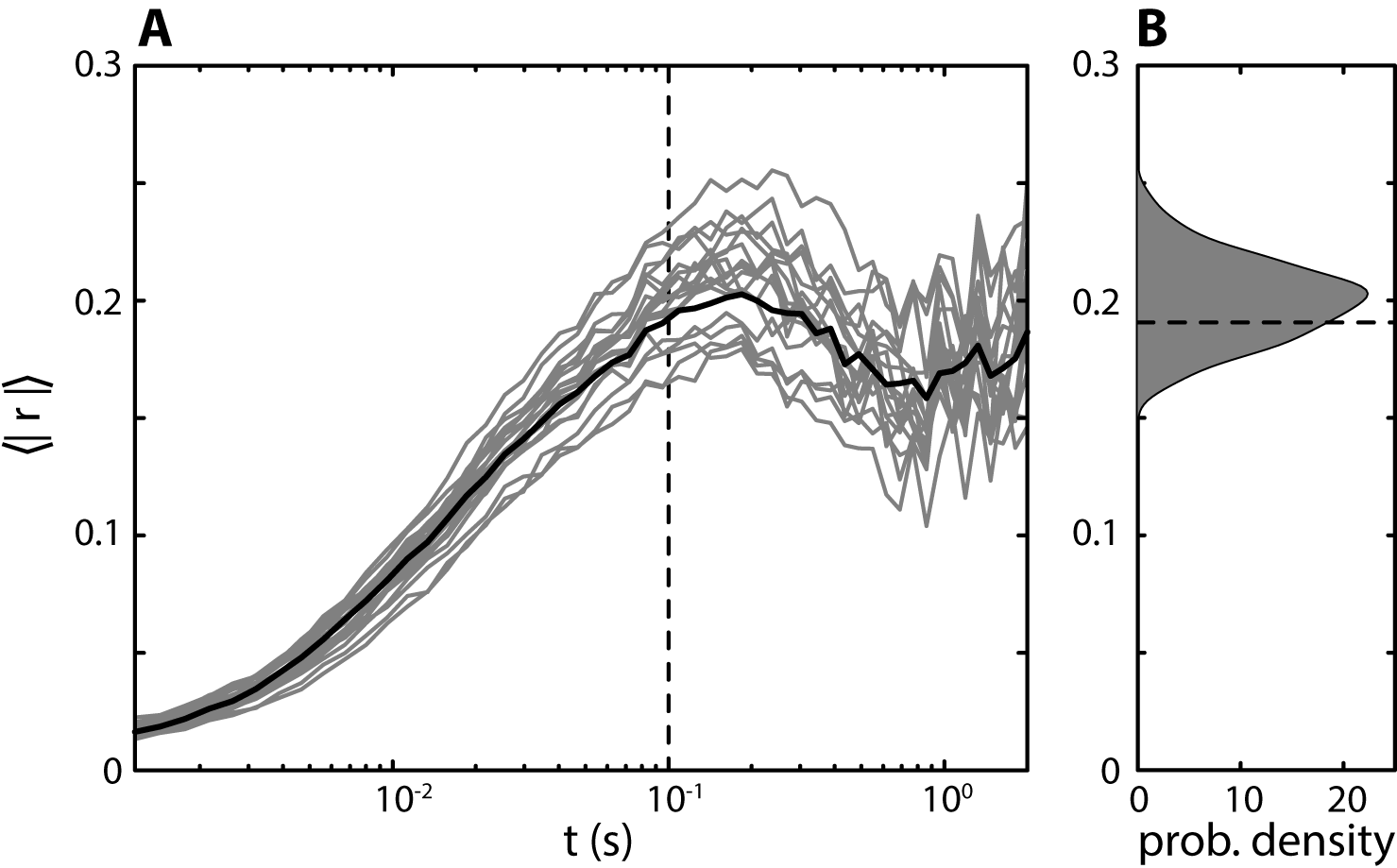

Supplement: S1 Fig — (A) Population-averaged absolute correlation coefficient for our entire CLS dataset (black line; N = 108) and for different sub-populations (gray lines; 20 random examples shown; each N = 17) generated by randomly selecting neuron pairs recorded in different animals. (B) Distribution of correlation coefficients obtained from independent sub-populations (gray area generated from 1010 different subpopulations) for a timescale of 100 ms (see vertical dotted line in (A)). The correlation coefficient obtained from the entire dataset (horizontal dotted line) was located within 42% of the area und the curve of the distribution, which is well below 95%. (TIF) [file pcbi.1005716.s001.tif]

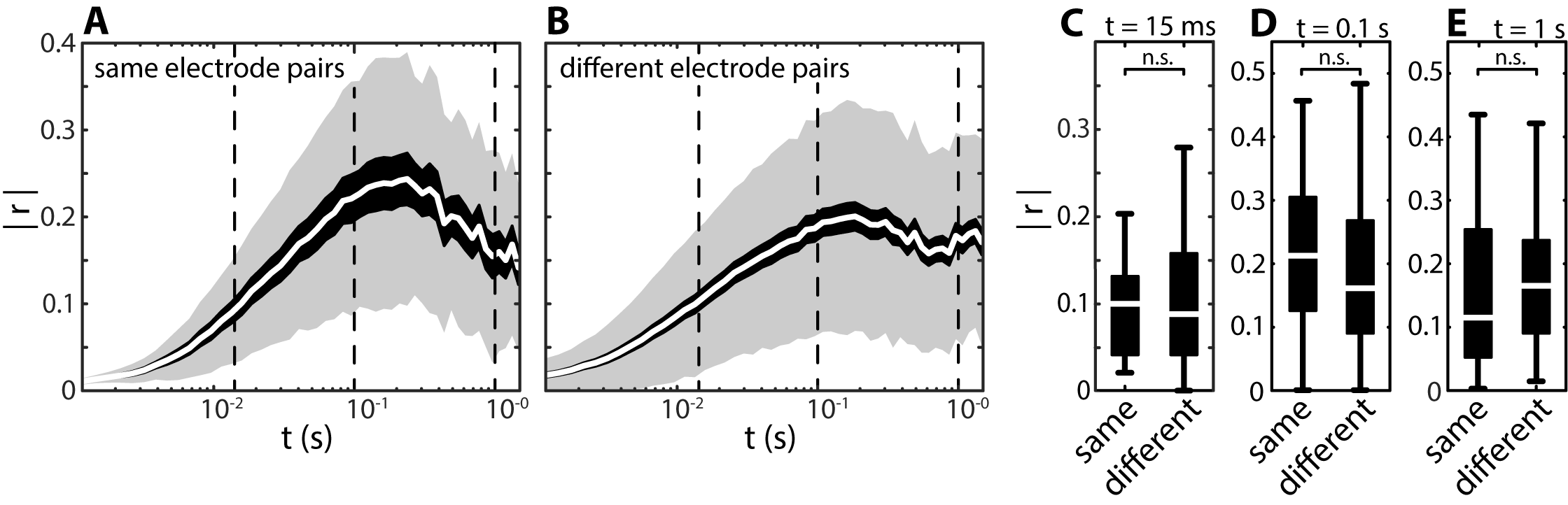

Supplement: S2 Fig — (A) Population-averaged absolute correlation coefficient for CLS pairs recorded on the same electrode (N = 26) as a function of time window. (B) Same as A, but for CLS pyramidal cell pairs that were recorded on separate electrodes (N = 82). In (A) and (B), shown are the mean (white line), SEM (black area) and STD (gray areas) of the population of pairs. (C–E) Population-averaged absolute correlation coefficients for same-electrode (“same”) and different-electrode (“different”) pairs in CLS at time windows (T) of 15 ms (C), 100 ms (D) and 1 s (E, see vertical dotted line in A & B). For each time window, the population-averaged absolute correlation coefficients obtained for same-electrode and different-electrode pairs were not significantly different from one another (Kruskal-Wallis: df = 1; Chi2 = 0/1.26/0.69/0.92; p = 0.95/0.26/0.34 for C/D/E). (TIF) [file pcbi.1005716.s002.tif]

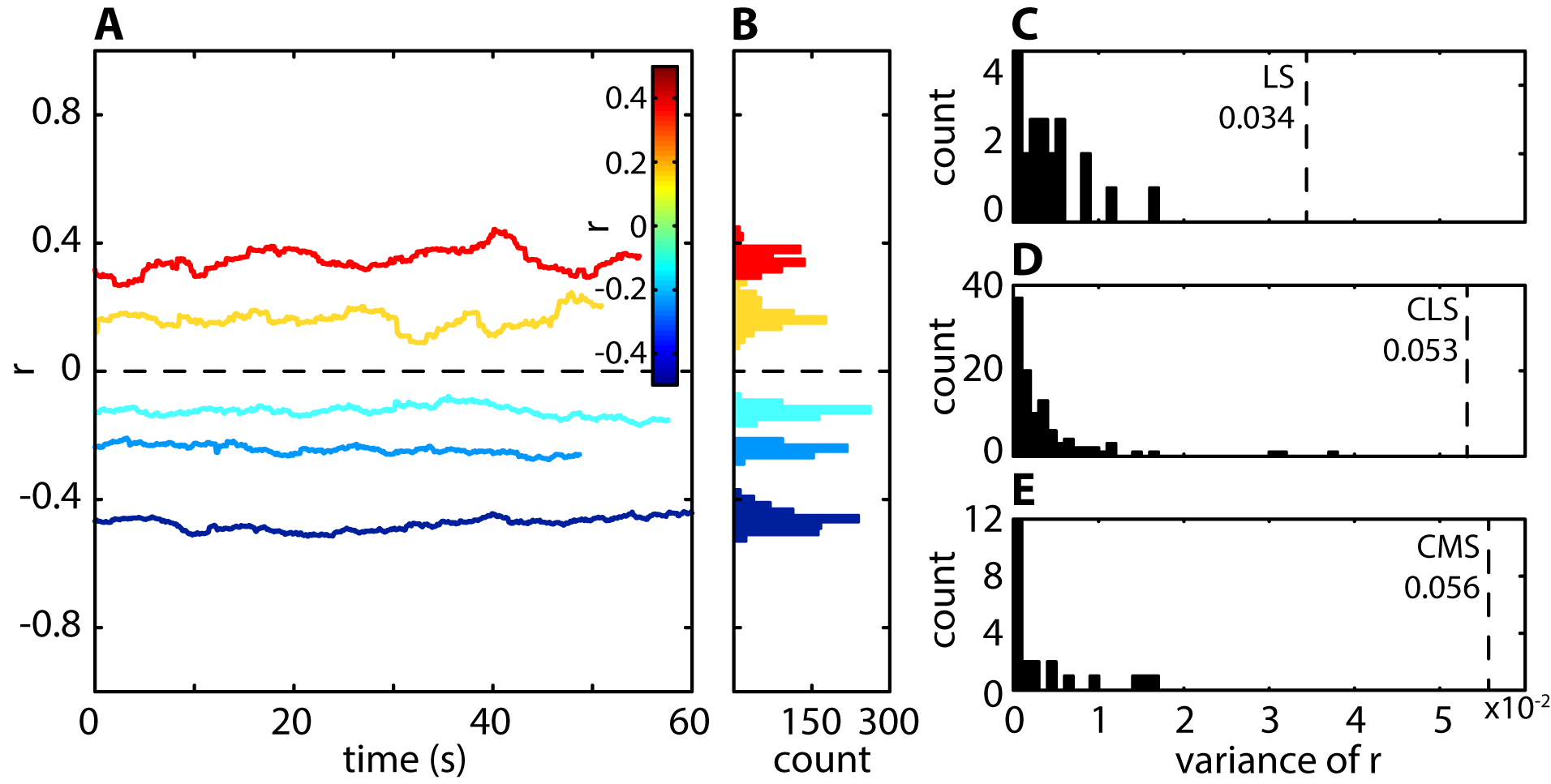

Supplement: S3 Fig — (A) Time varying spike count correlation coefficients (t = 100 ms, see Methods) from five example pairs. (B) Distributions of time varying correlation coefficients for the same five example pairs were generally unimodal. (C-E) Variance of the time varying correlation coefficients for all pairs in LS (C), CLS (D) and CMS (E). Variances of time varying correlation coefficients were generally small (mean for LS = 4.2 · 10−3; CLS = 3.8 · 10−3; CMS = 3.7 · 10−3) and lower than the variance of correlation coefficients across the population (LS = 3.4 · 10−2; CLS = 5.3 · 10−2; CMS = 5.6 · 10−2) by an order of magnitude. (TIF) [file pcbi.1005716.s003.tif]

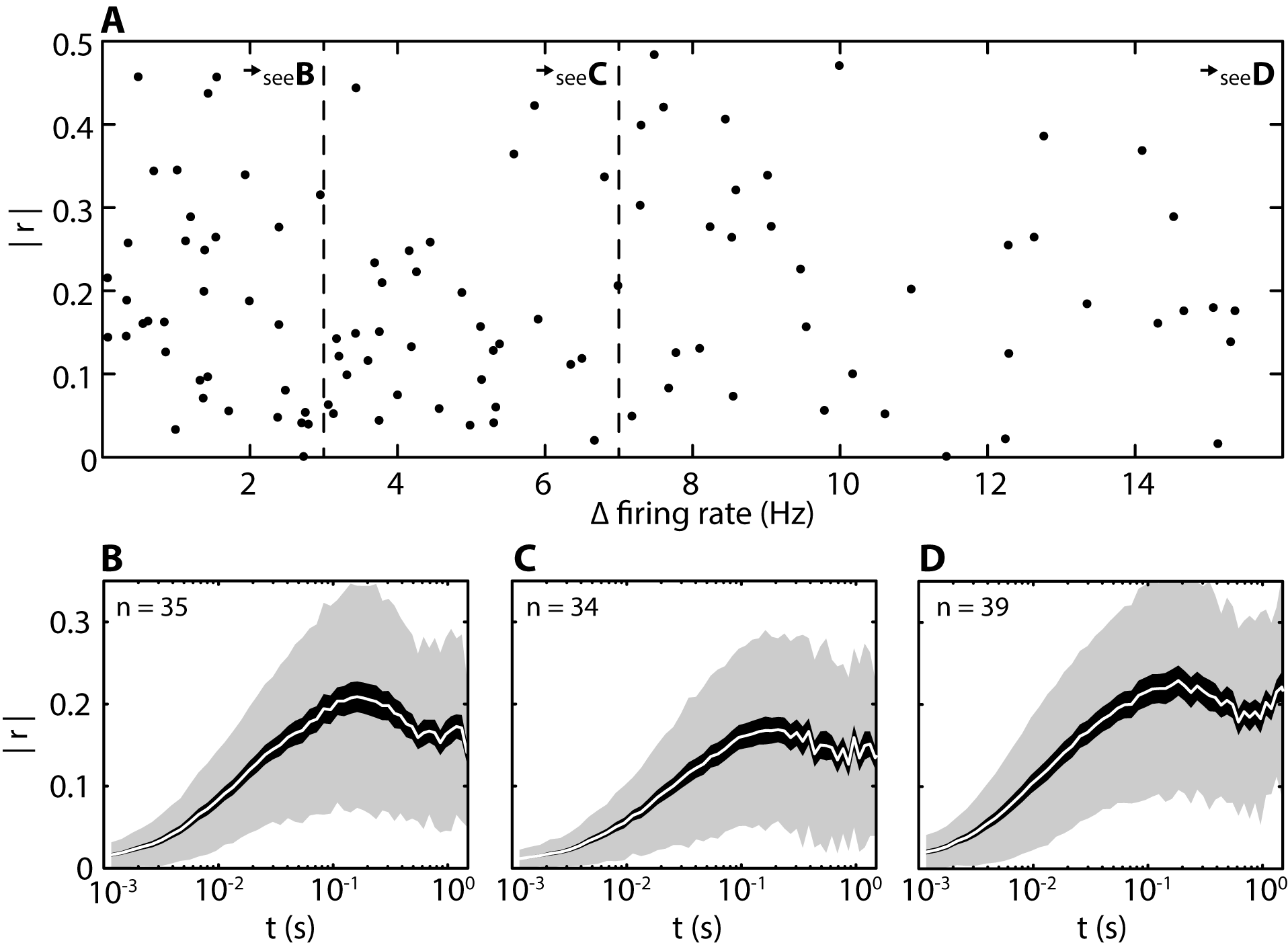

Supplement: S4 Fig — (A) Correlation coefficients (t = 100 ms) as a function of the difference in firing rate between neurons in each pair. (B-C) Pooling the data depending of different ranges of firing rate differences (see horizontal lines in A) showed similar means and variability in all cases (B: Δ firing rate 0–3 Hz; C: 3–7 Hz; D: >7 Hz). The means of the data were not significantly different when compared at various timescales (Kruskal-Wallis, t = 100 ms: df = 2; Chi2 = 3.88; p = 0.14; t = 1 s: df = 2; Chi2 = 0.49, p = 0.78). (TIF) [file pcbi.1005716.s004.tif]

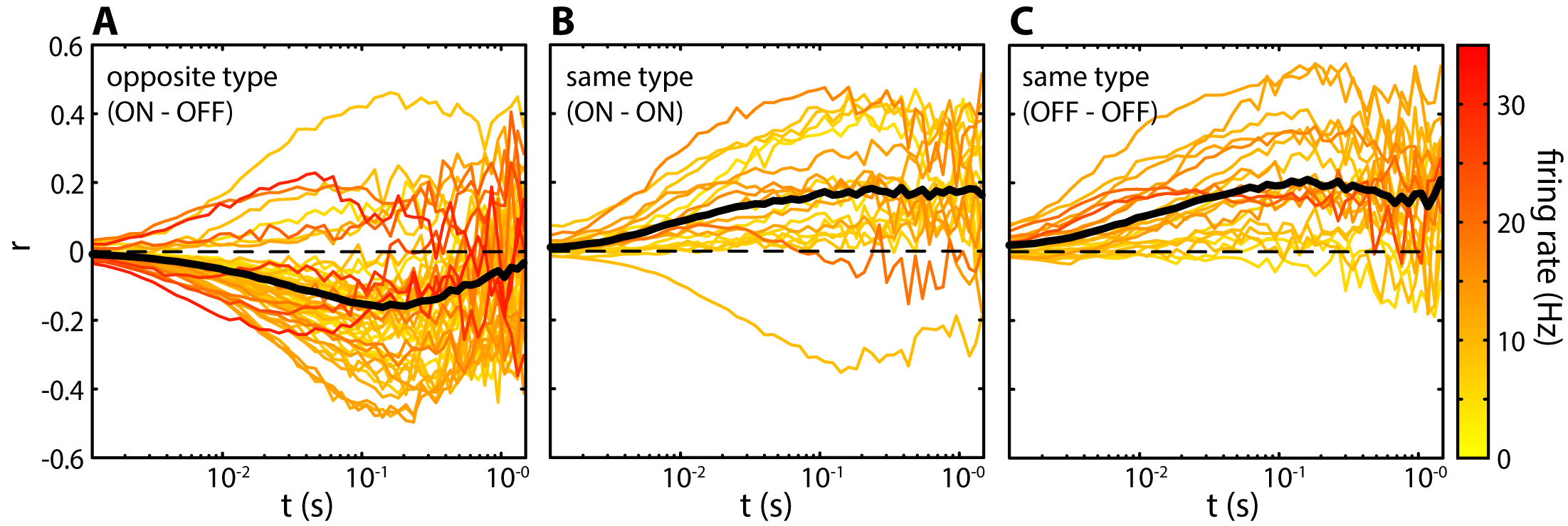

Supplement: S5 Fig — (A) Spike count correlations as a function of time window for opposite type (ON-OFF) pyramidal cell pairs (N = 48). (B & C) Same as (A), but for ON-ON (N = 20) and OFF-OFF (N = 22) pairs, respectively. In all panels, individual lines depict correlations of individual pairs, with the color showing the geometric mean of the firing rates for each cell in the pair. The black lines are the population averages. (TIF) [file pcbi.1005716.s005.tif]

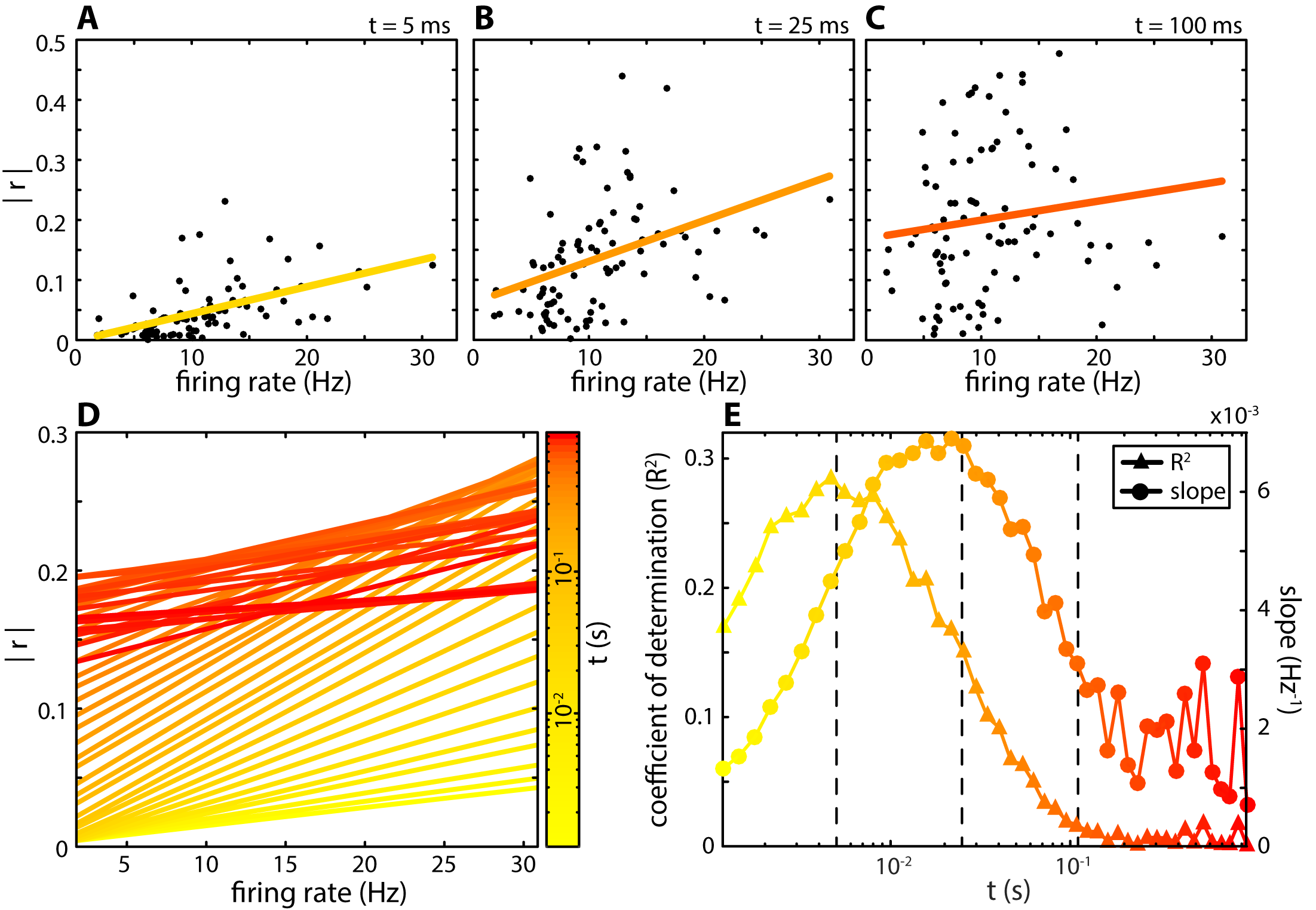

Supplement: S6 Fig — (A–C) Absolute correlation coefficient for individual pairs as a function of the geometric mean of the firing rates of each cell in the pair (black dots) for time windows of 5 ms (A), 25 ms (B), and 100 ms (C). Also shown are the best-fit straight lines. (D) Best-fit straight lines for different time window lengths (colored lines). The slope of the best-fit straight line first increased for time windows up to 25 ms and then decreased. (E) Goodness-of-fit R2 (triangles) and slope (circles) of the best-fit straight line as a function of time window. (TIF) [file pcbi.1005716.s006.tif]

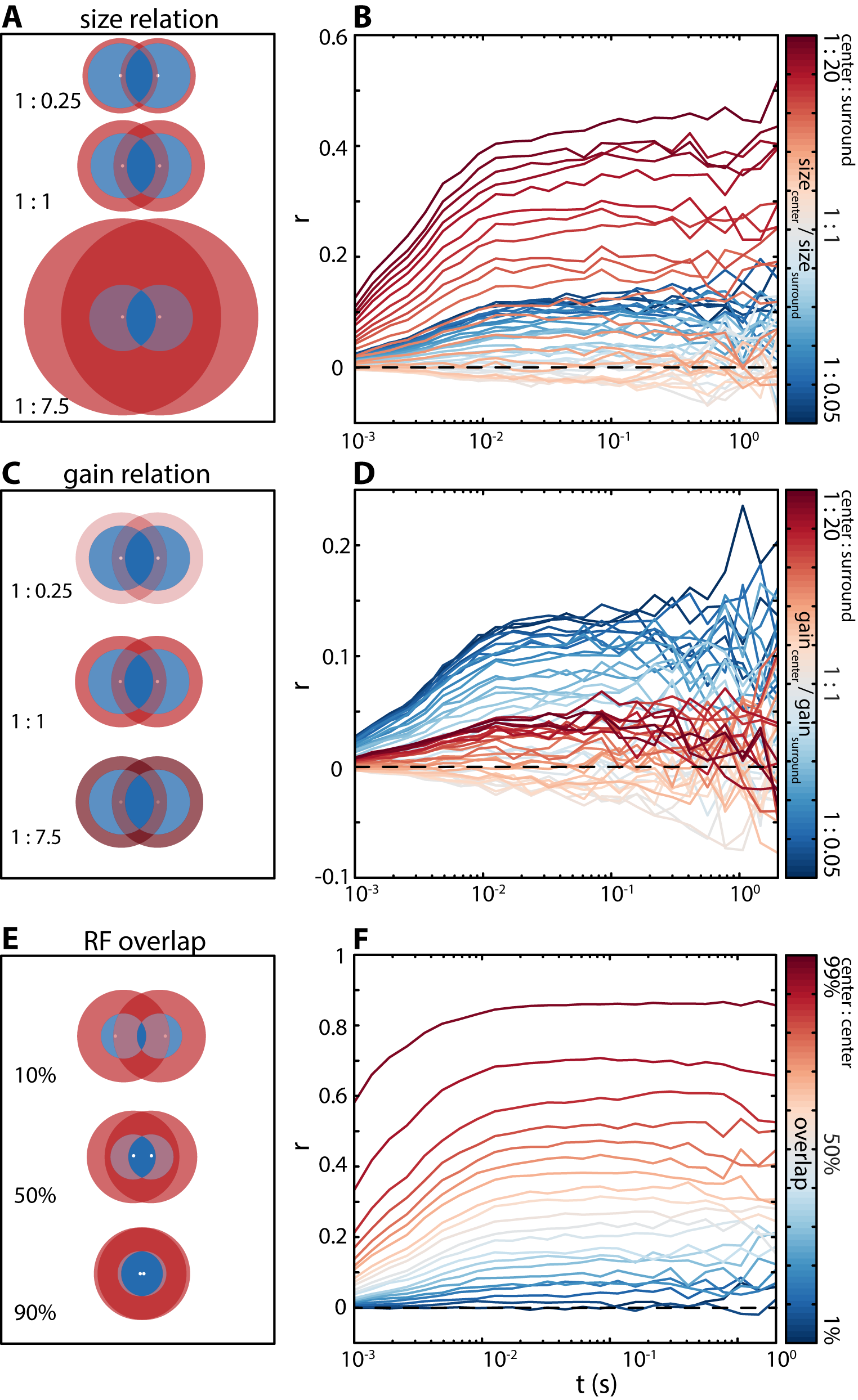

Supplement: S7 Fig — (A) RF centers (blue) and surrounds (red) for three different RF center-surround size values (1:0.25, top; 1:1 middle; 1:7.5 bottom). RF center overlap is that of CLS as per anatomical data, gain relation of center to surround was fixed at 1:1. (B) Correlation coefficient as a function of timescale for different relative surround size values (see color-code). (C) Same as (A), but when instead varying RF surround relative gain. (D) Same as (B), but for different relative surround gain values. (E) Same as (A), but sketched for different RF center overlaps. (F) Same as (B), but for different RF overlap values. Surround relative gain and size were 0.4 and 11.88, respectively. (TIF) [file pcbi.1005716.s007.tif]

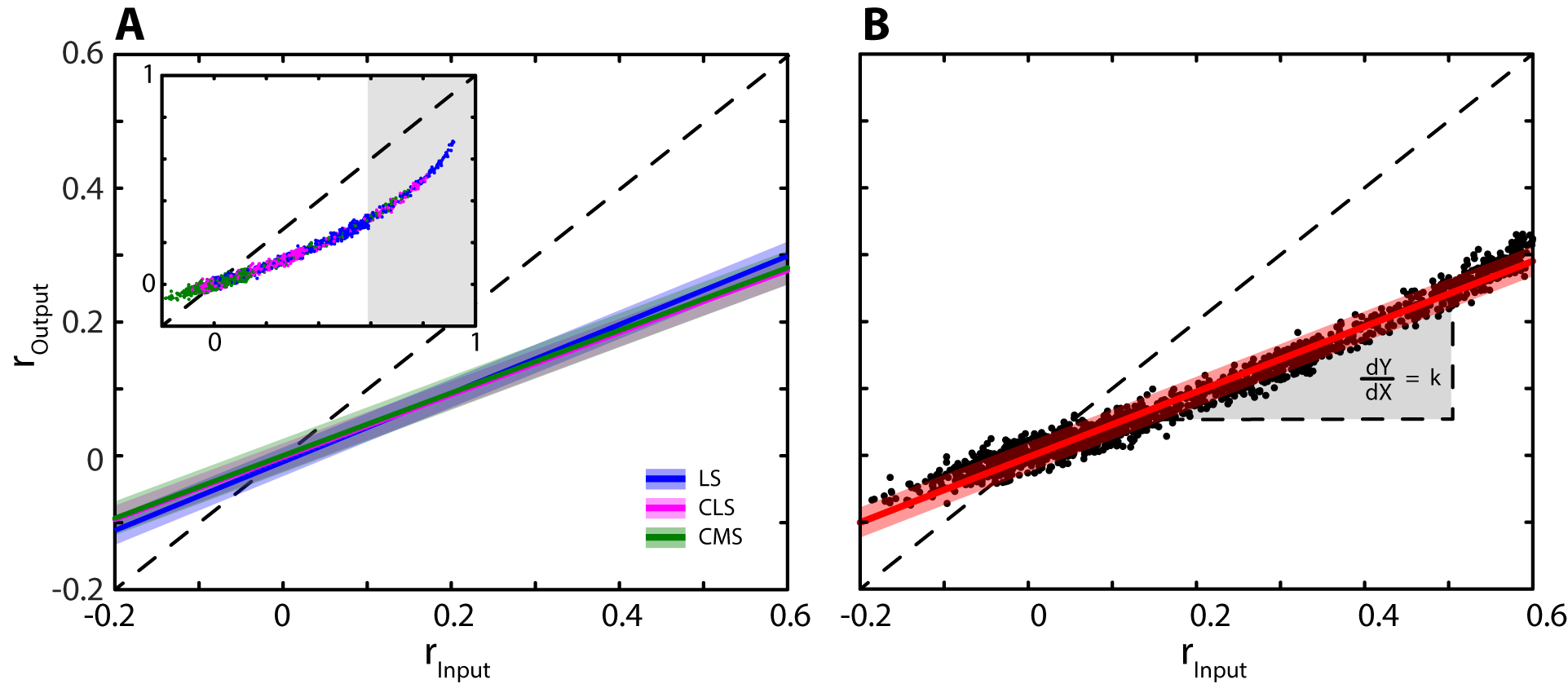

Supplement: S8 Fig — (A) Linear regressions obtained by linear least squares fit of output correlations as a function of input correlations for simulations mimicking the 3 segments (blue: LS; magenta: CLS; green: CMS). Shaded-areas represent one standard deviation of the data. Inset: Data from our simulations showing output as a function of input correlations. Our simulation results were similar to what was reported in earlier studies [11,71]. Importantly, the relationship between output and input correlation was linear in the range of physiologically observed correlation magnitudes (white area) based on our experimental data. (B) We fitted a linear regression on the pooled data from all segments in order to empirically obtain the scaling factor relating input to output correlations. We found k = 0.4884. (TIF) [file pcbi.1005716.s008.tif]

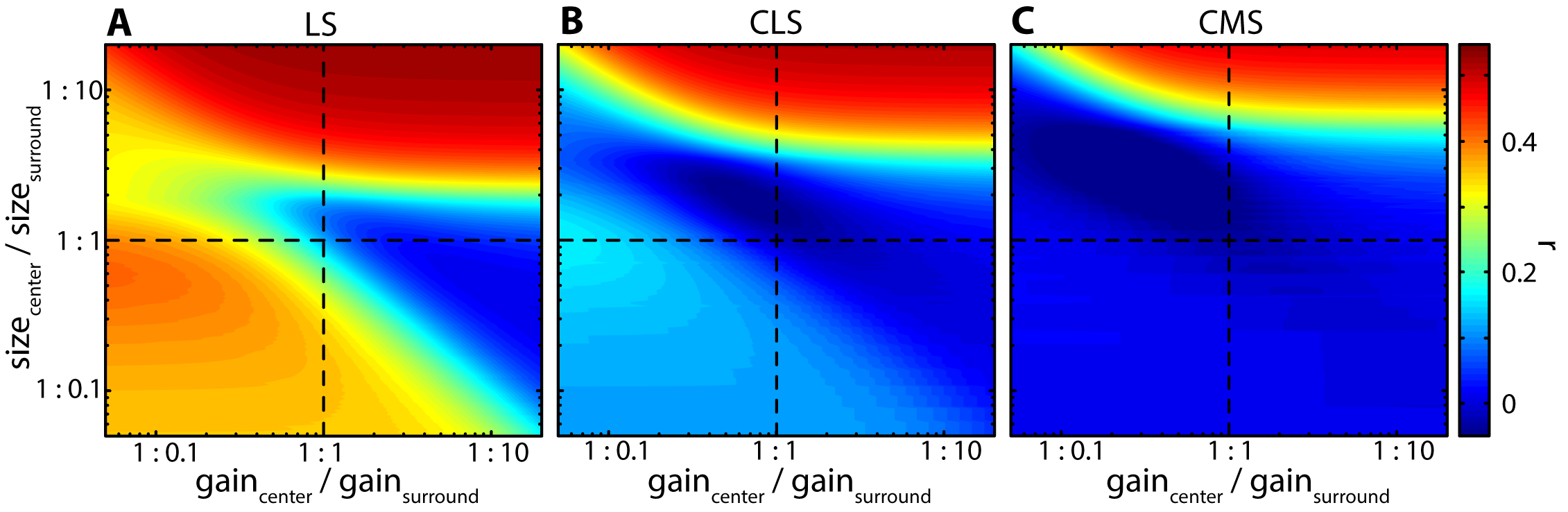

Supplement: S9 Fig — (A-C) Correlations obtained from our mathematical scale model (Eqs 15–20) for LS (A), CLS (B) and CMS (C) while varying RF balance in terms of RF center to surround gain relation and RF center to surround size relation. The results from our mathematical model were in good quantitative agreement with our numerical simulations (compare to Fig 5). (TIF) [file pcbi.1005716.s009.tif]

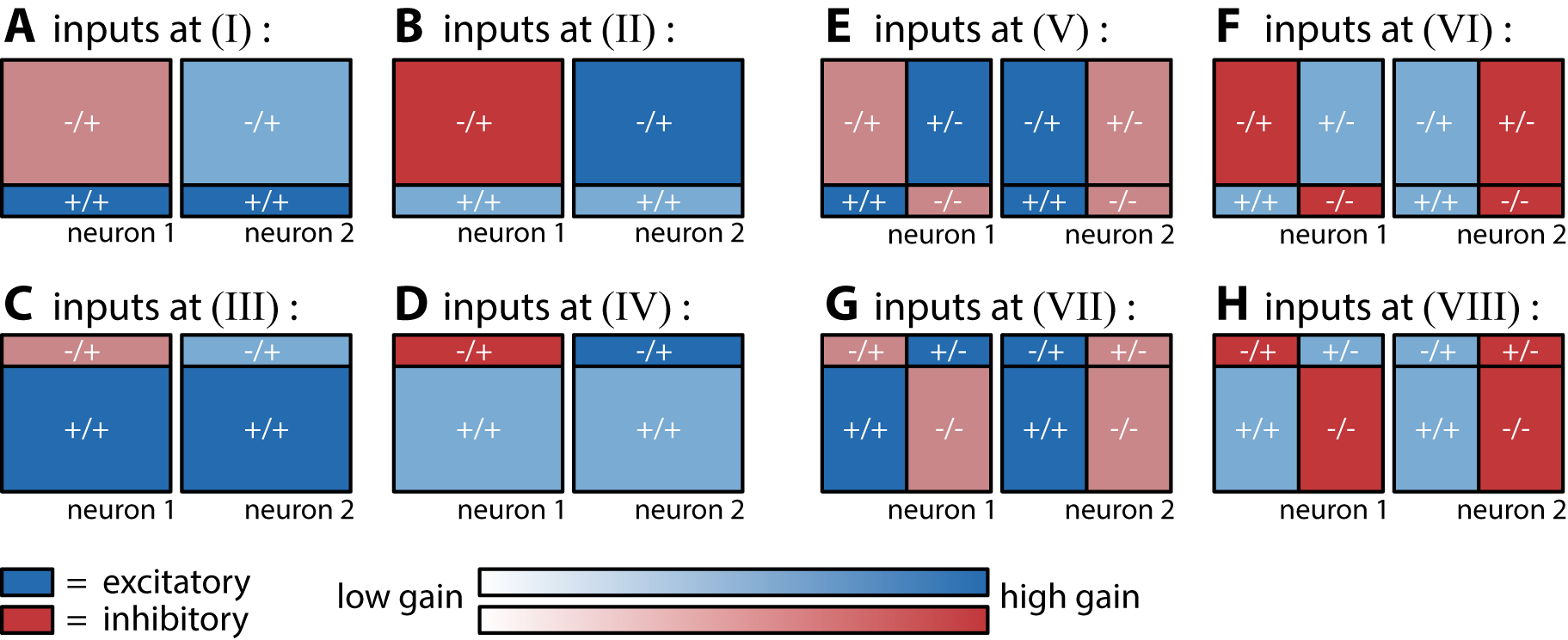

Supplement: S10 Fig — (A—D) In our theoretical model the relative amount of correlated (+/+) and anti-correlated (-/+) inputs was systematically varied in our model alongside the relative gain with which these inputs were mapped onto each respective neuron. Four (I–IV) or these input relations are visualized in the panel corresponding to the positions in Fig 6B. For each of the panels the left box visualizes how inputs are mapped onto neuron 1, while the right box visualizes the same inputs and how these are mapped onto neuron 2 (compare Fig 6A). Excitatory inputs are visualized in blue, inhibitory inputs in red, the saturation visualizes the strength of the gain. While the total amount of inputs was constant in all cases (A–D, total size of boxes) the relative amounts of input (horizontal divisions) was changed. (E- F) Same as (A-B) but for the model in which gains were mapped differentially according to the center vs. surround portion to which inputs belonged (compare Fig 6D). The panels correspond to the input relations (V–VIII) marked in Fig 6E. (TIF) [file pcbi.1005716.s010.tif]

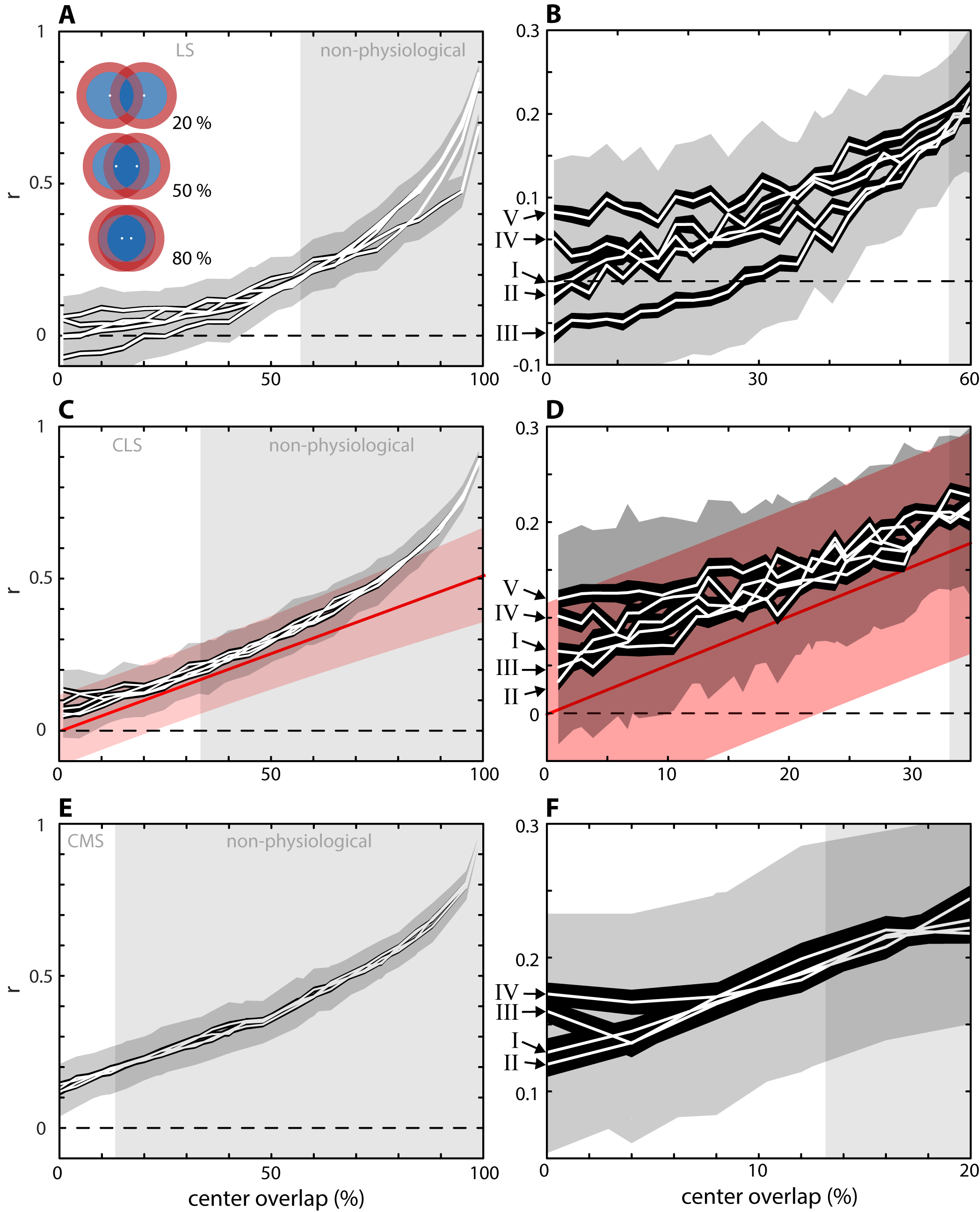

Supplement: S11 Fig — (A) Correlation coefficient as a function of RF center overlap (in percent) using different values of relative surround gain and size corresponding to the points I-V shown in Fig 5A. Shown are the mean (white line), SEM (black area) and STD (gray area). We used RF center values as per anatomical knowledge form LS. The gray shaded area marks RF center overlap not found anatomically and thus divides the data into physiologically realistic (white) and physiologically non-realistic (shaded gray) values of RF center overlap. (B) Same as (A) but magnified such as to better highlight the differences between the curves within the physiological relevant overlap range. (C & D) Same as (A & B), but for CLS RF center size values. The red line and shaded area depicts the linear fit of physiological CLS data previously published in [7]. (E & F) Same as (A & B), but for CMS RF center size values. (TIF) [file pcbi.1005716.s011.tif]

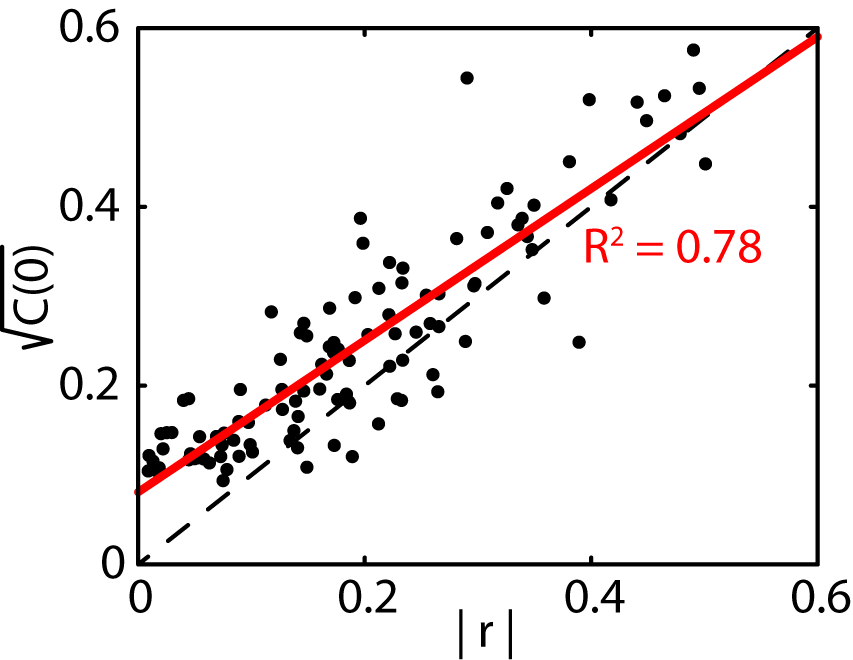

Supplement: S12 Fig — For all experimental CLS data we estimated correlation magnitude by taking the square root of the coherence between spike trains evaluated at frequency 0. There was good agreement between correlation estimates from the coherence and absolute correlation estimates from the spike count (red line is the best-fit straight line; R2 = 0.78; the dotted line shows the identity line). (TIF) [file pcbi.1005716.s012.tif]
